# Stool microbial composition is associated with recent and future diarrhea and fever events in breastfed Danish infants

**DOI:** 10.1128/msystems.00134-26

**Published:** 2026-05-11

**Authors:** Laurynne C. Coates, David H. Storms, Sarah S. Spearman, Setareh Shahab-Ferdows, Sophie Hilario Christensen, Jack I. Lewis, Christian Mølgaard, Kim F. Michaelsen, Lindsay H. Allen, Danielle G. Lemay, Mary E. Kable

**Affiliations:** 1 U.S. Department of Agriculture–Agriculture Research Service, Western Human Nutrition Research Center 57738, Davis, California, USA; 2 Department of Nutrition, University of California, Davis 115100, Davis, California, USA; 3 Department of Nutrition, Exercise, and Sports, University of Copenhagen 87071, Copenhagen, Denmark; University of Southampton, Southampton, United Kingdom; The University of Melbourne, Melbourne, Australia; UK Centre for Ecology & Hydrology, Wallingford, United Kingdom

**Keywords:** gut microbiota, diarrhea, fever, infant, *Granulicatella*, *Haemophilus*, logistic regression, random forest model

## Abstract

Gastroenteritis is an important cause of ailment among infants in high-income countries, including Denmark. There are many cases of diarrhea among Danish children for which no etiological agent is detected. This study investigated the associations between gastroenteritis-related morbidities (diarrhea, fever, and vomiting) and gut microbial community in well-nourished, breastfeeding Danish infants. Infant stool samples, morbidity questionnaires, and diet/breastfeeding questionnaires were collected at three time points during the first 8.5 months of life. The V4-V5 region of the 16S rRNA gene was amplified from stool DNA extracts, sequenced with Illumina MiSeq, and analyzed using QIIME2. PERMANOVA, linear mixed-effects modeling, and ANCOM-BC2 were used to identify associations between infant gut microbiome and gastroenteritis-related morbidities across all visits. Logistic regression and random forest models were employed to determine whether early gut microbial alpha diversity or abundance, respectively, could predict morbidity later in infancy. Alpha diversity was negatively associated with diarrhea. *Granulicatella* abundance was positively associated with diarrhea. From 3.5 to 6 months of age, Bacteroidales abundance was negatively associated with fever. Faith’s phylogenetic diversity, Staphylococcales abundance, and *Haemophilus* abundance during 3.5 months of life were positively associated with future diarrhea during ages 3.5–8.5 months. Pielou’s evenness and Actinobacteriota abundance within 3.5 months of life had negative and positive associations, respectively, with fever during ages 3.5–8.5 months. This study reveals diarrhea is associated with stool *Granulicatella* in well-nourished, breastfeeding Danish infants, as it is in children from low-income countries, and that the early gut microbiome may contribute to risks of diarrhea and fever morbidities later in infancy.

**CLINICAL TRIALS:**

This trial was registered at ClinicalTrials.gov as NCT03254329.

**IMPORTANCE:**

Gastroenteritis continues to cause much morbidity among infants in high-income countries, and the relationship with the gut microbiome is not fully understood, especially for well-nourished and breastfeeding infants. In the study presented here, infant stool Staphylococcales abundance (comprised of *Staphylococcus* and *Gemella*) and *Haemophilus* abundance during the first few months of life were positively associated with later diarrhea in well-nourished and breastfeeding Danish infants. Meanwhile, the abundance of *Granulicatella* (a facultative anaerobe with pathogenic potential) was greater in stool from infants who had recent diarrhea, suggesting further research is needed to determine its possible role in diarrhea and recovery from diarrhea. Fever usually did not co-occur with diarrhea or vomiting. Early life Actinobacteriota abundance was positively associated with later fever. This phylum was represented here by both pathobionts (*Actinomyces*) and mutualists (bifidobacteria), which may have contributed to fever differently—pathobionts through infection and mutualists through promotion of effective immune response to infection.

## INTRODUCTION

During birth and infancy, the gut is colonized by microbes that play critical roles in shaping the health of the individual, particularly with respect to immunity and metabolism (1). Mutualistic bacteria in the infant gut, such as bifidobacteria, *Bacteroides,* and *Prevotella*, degrade undigested glycans from milk (oligosaccharides) or complementary foods (2–4) and, in turn, produce short-chain fatty acids that feed intestinal cells and modulate immunity (5). Mutualistic bacteria can also enhance colonization resistance to enteric pathogens (6). Meanwhile, pathogenic and non-mutualistic microbes in the gut can cause local and systemic inflammation and can contribute to a dysbiotic gut microbial community, which has been linked to a wide range of acute and chronic diseases (1, 7).

Diarrhea is the second leading cause of death and a leading cause of malnutrition and morbidity among children under 5 years old worldwide and can be accompanied by vomiting and fever (8), which are also common morbidities in infancy (9–11). Causative agents include bacteria (e.g., certain *Escherichia coli* strains, *Shigella*), viruses (e.g., rotavirus, norovirus), and parasites (e.g., *Cryptosporidium*) (11, 12). Diarrheal episodes and diarrhea-related deaths afflict children in low-income countries to a greater extent than those in high-income countries, largely because of a lack of sanitary water, hygiene, and access to/use of the rotavirus vaccine (12). However, diarrhea in infants may also occur as a symptom of non-communicable diseases, such as allergies, that cause intestinal inflammation and damage, or as nutrient malabsorption (13). Moreover, a systematic review of global diarrheal episodes estimated that about 34% of diarrheal episodes did not have a known etiology (14).

A previous study of Danish children showed 46% of diarrhea cases in children were not associated with any known gastrointestinal pathogen (15). This could suggest the etiological pathogen was not yet detectable, that the diarrhea was a symptom of a non-communicable host illness, or that there were other pathobionts causing diarrhea. Importantly, among the matched control stool samples collected in that same study from children who did not have any diarrhea or abdominal pain with fever in the month prior to sample collection, a potential diarrheal pathogen was detected in 22% of samples (15). These control samples containing diarrheal pathogens could be a result of detection during the incubation phase of infection, a long shedding window after symptomatic infection (16), or asymptomatic infection resulting from pathogen tolerance (17). When exposed to an enteric pathogen, the resident mammalian gut microbial community interacts with the pathogen and modulates host immunity to influence colonization resistance and tolerance to pathogens (6, 18).

Bifidobacteria are gut mutualists, and some strains, when used as probiotics, have been shown to reduce diarrheal episodes in infants (19), lessen the severity, incidence, and duration of rotavirus-induced diarrhea (20), and lessen the symptoms of enterotoxigenic *E. coli*-induced diarrhea in murine models (21). Among Malawian infants, *B. bifidum* and *B. pseudolongum* were negatively associated with diarrhea at 6 months (22). Bifidobacteria are adapted to degrading oligosaccharides in the breast milk and are enriched with breastfeeding (23). Breastfeeding is protective against diarrhea (24), and breast milk oligosaccharides can reduce infectivity of gastrointestinal pathogens (23). Meanwhile, gut bacterial taxa, other than known diarrhea-causing pathogens, have been positively associated with diarrhea. *Streptococcus* and *Granulicatella* were associated with diarrhea among children under 5 years of age in low-income countries (25), while *Prevotella* was associated with diarrhea among children aged 1–6 years in Denmark (26). In the latter publication, authors reasoned that *Prevotella* may have been associated with diarrhea because a study of microbial succession following *V. cholerae*- and ETEC-caused diarrhea found *Prevotella* enriched during the late stage recovery from diarrhea (27).

The microbes in the gut that affect a child’s susceptibility to diarrhea and the impact of diarrhea on the composition of the infant gut microbial community are not fully understood, especially within discrete geographical locations. The Mothers, Infants, and Lactation Quality (MILQ) Study is a study of mother–infant dyads in four countries aimed at developing human milk nutrient reference values from 1 to 8.5 months of life (28). It was leveraged here to fulfill a secondary objective: to determine the gut microbes associated with diarrhea and related morbidities—fever and vomiting—in well-nourished, breastfeeding Danish infants.

## MATERIALS AND METHODS

### Experimental design and inclusion and exclusion criteria

Written informed consent was obtained from all mothers who participated in the study. As part of the MILQ study, stool samples, questionnaires regarding maternal and infant morbidity (which included questions regarding medicine and supplement use), and infant breastfeeding complementary feeding practices were collected at three postpartum time points: ~1–3.49 months (visit 2), ~3.5–5.99 months (visit 3), and 6.0–8.49 months (visit 4). Visit 1 involved colostrum sample collection and newborn screening 24–72 h after birth. Maternal sociodemographic information was collected once, during visit 2. Details regarding the inclusion and exclusion criteria for mothers and infants in the MILQ study were reported by Allen et al. (28). In brief, Danish mother–infant dyads were enrolled in the study if maternal age was 18–40 years, maternal BMI was 18.5–30.0 prepregnancy, there were no maternal medical problems (e.g., gestational diabetes or pre-eclampsia), the mother did not smoke, the mother consumed <50 mL alcohol per day, the pregnancy was full term (37–42 weeks of gestation), and the birth was singleton. And, in brief, mother–infant dyads were excluded if infants were not at least partially breastfed during the first 6 months of life. Ultimately, 109 mother–infant dyads met these criteria and had a stool sample collected at all three timepoints in the study. Among these 109 infants, 32.1% had diarrhea, 59.6% had fever, and 25.7% had vomiting during 1–3 timepoints.

### Stool sample and questionnaire collections

Stool samples and questionnaires were collected once per visit at various ages within each visit period. Mothers chose when (date and time) to collect a stool sample for a visit period and when to come in for a visit (to complete the interview and submit the stool sample), so long as it complied with the age range for that particular visit period. Within each visit, there was representation of the full range of infant ages at stool collection (Fig. S1), and the time at stool collection ranged from 12:45 am to 10:34 pm. Usually, 2–3 months passed between subsequent stool samples from the same infant (Fig. S2). Collection took place at home by the parents, where plastic wrap was used to line the diaper, and when the infant had passed a bowel movement, about 1 g of the stool was scooped up and placed in a tube. Stool was stored in home freezers at −18°C and then transferred to −70°C until being processed. Questionnaires were completed by trained staff members during interviews with mothers. Diarrhea was defined as three or more watery stools in a day, which is in agreement with the World Health Organization definition of diarrhea (11). The dates at which infant diarrhea, fever, or vomiting occurred were not recorded. Instead, mothers were asked, for each morbidity separately, if the morbidity occurred within the week prior to the visit, and if the morbidity occurred anytime since the previous visit interview (excluding the week prior to the visit interview).

### Bacterial DNA isolation and sequencing

For each stool sample, 100 mg of frozen stool was used for DNA extraction using the ZymoBIOMICS MagBead-96 DNA kit (cat # D4308) and the Eppendorf epMotion liquid handler 5075t. The manufacturer’s instructions were followed with the exception that two rounds of elution were carried out—40 µL of DNase/RNase-free water was used for the first round and 25 µL of DNase/RNase-free water for the second. Two-step elution was carried out to maximize DNA yield. Negative control (aka blank) samples and ZymoBIOMICS microbial community standard samples (cat # D6300) were included in each stool extraction batch to confirm negligible environmental contamination. Isolated DNA was sent to the Integrated Microbiome Resource at Dalhousie University (Halifax, Nova Scotia, Canada) for PCR and library preparation according to the methods reported by Comeau et al. (29), and paired-end 300 base sequencing on the Illumina MiSeq platform. Forward primer 515FB (GTGYCAGCMGCCGCGGTAA) and reverse primer 926R (CCGYCAATTYMTTTRAGTTT) (30) were used in 25 cycles of PCR to amplify the V4-V5 region of the 16S rRNA gene.

### Sequence processing

Demultiplexed reads were imported into QIIME2 version 2022.11 (31), where the forward and reverse primers were trimmed from forward and reverse reads with cutadapt (32). DADA2 (33) was then used to truncate reads (at 222 bases for forward reads and at 171 bases for reverse reads), to denoise and dereplicate reads, and to filter reads of chimeras. Truncation lengths were chosen based on patterns in quality score distributions among the reads (50th percentile ≥ Q30) and as well to ensure sufficient overlap (12 bp minimum) for merging of paired reads. Amplicon sequence variants (ASVs) that had only one count in the entire data set (singletons) were filtered from the resulting feature table and representative sequence document. The SILVA 138 SSURef NR99 database (34), which was preformatted with RESCRIPt and made available as QIIME2 artifacts for full-length sequences and taxonomies (35), was used for taxonomic classification of ASVs. Simulated amplicon reads were extracted from the reference database and used to create a scikit-learn naïve Bayes classifier for reads. The classifier was then used to classify reads by taxonomy. Features identified as mitochondria, chloroplasts, or eukarya were removed with the script wrapper “qiime taxa filter-table.” Among stool samples, there were 22,637,580 total paired-end reads (median 33,739) before processing and filtering. After processing and filtering, there were 13,833,996 total paired-end reads (median 21,523). See the GitHub repository at https://github.com/L-Coates/MILQ-Denmark for QIIME2 commands used for sequence processing, taxonomic classification, and sequencing filtering.

### Infant stool batch effect assessment

There was no difference in DNA concentration by extraction batch (ANOVA with box-cox transformed DNA concentration, *P*-value > 0.05). There was a difference in read depth by DNA extraction batch and PCR batch, but not by sequencing batch (Wilcoxon rank sum test). To determine whether any of these covariates were associated with microbial community structure, permutational analysis of variance (PERMANOVA) using unweighted and weighted UniFrac distance matrices (function adonis2 from vegan package v. 2.6.4) was performed. None of the aforementioned covariates were associated with beta diversity among the infant stool samples. Therefore, the DNA extraction batch, the PCR batch, and the sequencing batch were not included in subsequent models involving morbidity outcomes.

### Stool sample selection

After quality control steps, including removal of reads assigned to chloroplast, mitochondria, or eukarya taxonomy, the lowest read depth among the ZymoBiomics mock control samples was 6,886. Any stool sample below this read depth was excluded from the analysis. Among the stool samples with ≥6,886 reads, only those that were a part of a complete sample set for an infant (i.e., one stool sample from all three visits) were selected for analyses. This resulted in 327 stool samples collected from 109 infants that were used in subsequent analyses. Among these 327 stool samples, the read depth range was 6,963–123,521, with a median of 24,750 and a mean of 27,549. There were 1,782 ASVs and 244 genera represented among these reads.

### Infant stool microbial diversity and differential abundance analyses with infant morbidity

Using QIIME2, beta diversity and alpha-diversity metrics were calculated from the feature table rarefied without replacement at 6,963 counts (the minimum read depth among the set of stool samples analyzed). Beta-diversity metrics—unweighted and weighted UniFrac distances—were both tested separately for association with infant morbidity using PERMANOVA. Morbidity (diarrhea, fever, and vomiting) was set as a fixed effect on the response variable, beta diversity. In addition, the covariates—infant, age at stool collection, antibiotic use, probiotic use, laxative use, gender, mode of delivery, mother’s parity (called “first pregnancy” with values “yes” and “no”), number of people in the household (called “household size” with values “≤3” and “>3”), and exclusive breastfeeding in first 4 months (80/109 infants total)—were included as fixed effects (see Table S1 for statistical model parameters). Follow-up reduced PERMANOVA models were also performed, which did not include covariates that were not significantly associated with beta diversity in the initial (full) models (see R code in GitHub repository https://github.com/L-Coates/MILQ-Denmark). Similarly, each visit was also analyzed separately for the association between beta diversity and morbidity using PERMANOVA. In these non-longitudinal models, the infant was not included, but all other covariates previously mentioned for the full models were included here as well. Follow-up reduced PERMANOVA models were also performed for the non-longitudinal (cross-sectional) subsets of data that did not include covariates that were not significantly associated with beta diversity in the initial models. Alpha-diversity metrics—Shannon entropy, Faith’s phylogenetic diversity, total observed ASVs (which were box-cox transformed), and Pielou’s evenness—were each separately tested for association with infant diarrhea, fever, and vomiting using linear mixed-effects modeling. Alpha diversity was set as the response variable, while infant was set as the random effect, and morbidity was set as a fixed effect, and the following covariates were set as additional fixed effects: age at stool collection, antibiotic use, probiotic use, laxative use, gender, mode of delivery, mother’s parity, number of people in the household, and exclusive breastfeeding in first 4 months. Follow-up reduced linear mixed-effects models were also performed with one morbidity type (diarrhea, fever, or vomiting) at a time and without inclusion of covariates that were not significantly associated with alpha diversity in the initial models.

Differential abundance of infant stool microbes (ASVs, species, genera, families, orders) with diarrhea, fever, or vomiting during 0.8–8.5 months of age (i.e., using longitudinal, repeated measures data set) was determined with analysis of compositions of microbiomes with bias correction 2 (ANCOM-BC2) (36) with individual set as a random effect, morbidity set as a fixed effect, and the following covariates set as fixed effects for the full models: age at stool collection, antibiotic use, probiotic use, laxative use, gender, mode of delivery, mother’s parity, number of people in the household, and exclusive breastfeeding in the first 4 months. Follow-up reduced ANCOM-BC2 models were also performed, which did not include covariates that were not significantly associated with stool microbes in the initial full models (listed in Table S1). Differential abundance of infant stool microbes (ASVs, species, genera, families, orders) with morbidity in each separate visit (i.e., splitting repeated measures into separate cross-sectional data sets by visit) was determined with ANCOM-BC2 with morbidity set as a fixed effect, and the following covariates set as fixed effects for the full models: age at stool collection, antibiotic use, probiotic use, laxative use, gender, mode of delivery, mother’s parity, number of people in the household, and exclusive breastfeeding in first 4 months. A significant association (FDR-adjusted *P*-value < 0.05) between a stool microbial taxon and a morbidity, or other covariate of interest such as probiotic use, was followed up with a Wilcoxon rank-sum test performed on rarefied counts of the taxon. Stool microbial abundance correlations (network analysis) were determined using the sparse inverse covariance estimation for ecological association inference (SPIEC-EASI) (37) method and visualized with NetCoMi (38) package in R. Only genera with a mean relative abundance ≥0.001% were included in network analysis. Correlation significance was set at alpha < 0.05 and local FDR < 0.05.

To explore potential relationships between early infant gut microbiome and later morbidity outcomes, stool microbial alpha diversity and microbial abundance from visit 2 (first 0.8–3.49 months of life) were related to diarrhea, fever, and vomiting outcomes in visits 3 and 4 using logistic regression and random forest modeling, respectively. Since gut microbe composition varies with age, especially in early life, age at time of visit 2 stool collection was first explored as a potential confounding variable in the prediction of morbidity outcomes in visits 3 and 4. No difference in age at visit 2 stool collection was found between morbidity outcome groups (e.g., infants with diarrhea in visits 3 or 4 vs. infants without diarrhea in visits 3 and 4) (*P*-value > 0.05 for Wilcoxon rank sum test and t-test with ordered quantile normalization transformed values). Logistic regression was employed to determine whether any of the four stool microbial alpha-diversity measures from visit 2 were predictive of diarrhea, fever, or vomiting reports in visits 3 and 4. For each morbidity outcome, the data were split into training (70%) and test (30%) sets. Since the minimum number of events among the response variables ranged from 25 to 46, models were fit on the training set with only three predictor variables to avoid a low number of events per variable. The models only included three variables: alpha-diversity metric, household size, and morbidity in visit 2 (i.e., morbidity from birth to 3.49 months of age). For example, the model relating Shannon entropy in stool samples from visit 2 to diarrhea in visits 3 or 4 followed the formula “diarrhea in visits 3 or 4 ~diarrhea in visit 2 + household size + Shannon entropy.” Diarrhea in visit 2 was included as a variable to account for the possibility that prior diarrhea could increase the likelihood of subsequent diarrhea events (11). Household size was included as a variable because infants living in larger households may have greater exposure to infections causing diarrhea, fever, and/or vomiting. In this cohort, it is unknown whether household size largely captures infant exposure to infectious illnesses because information was not gathered from mothers regarding infants’ enrollment in daycare, travel frequency, or morbidity among household members. Balanced accuracy, F1-score, and area under the receiver operating characteristic curve (ROC AUC) were calculated for each fitted logistic regression model that determined alpha diversity in visit 2 to be significantly associated with morbidity in visits 3 and 4 (*P*-value < 0.05). In the follow-up, a Wilcoxon or *t*-test was performed to determine whether the alpha-diversity metric differed between the morbidity outcome groups.

Random forest models were designed to use stool microbial taxonomic abundances (rarefied counts) in visit 2 as predictors of reported morbidity in visits 3 and 4. TaxaHFE (39)—an algorithm for collapsing hierarchical features which involves permuting random forest models and dropping lower “descendent” taxa if the mean importance of the higher “parent” taxon is greater—was employed with rarefied taxonomic counts (at all taxonomic levels from kingdom to species) to select the taxa most likely to predict morbidity outcome in visits 3 or 4 and consequently to use as features (a.k.a. predictors) in random forest models. If any of these taxa had an absolute correlation of 0.9 or greater, then one of the taxa among the correlated pair was removed from the feature set to be included in the random forest model. The order Frankiales was removed from the feature set used in the random forest model to predict vomit outcome in visits 3 or 4 because it was highly correlated with Flavobacteriales and an uncultured species of Bacteroidetes. The following is an example of a feature set used in the random forest model for diarrhea outcome in visits 3 or 4: infant diarrhea in visit 2, household size, and rarefied counts for *Bacteroides vulgatus*, *Bacteroides uniformis*, Corynebacteriales, Flavobacteriales, *Haemophilus*, *Prevotella timonensis*, *Rhodoferax*, *Sutterella wadsworthensis*, and Staphylococcales abundances in visit 2. Meanwhile, the random forest model for fever outcome in visits 3 or 4 included the predictors: infant fever in visit 2, household size, and microbial rarefied counts from visit 2 stool for Actinobacteriota, Bacilli, *Bacteroides fragilis*, *Negativicutes*, and an unknown human gut species of *Clostridium sensu stricto 1*. For each morbidity outcome, the data were split into training (70%) and test (30%) data sets. The performance of a random forest model in predicting the outcome in the test data set was assessed with calculations of accuracy, balanced accuracy, F1-score, and ROC AUC. Permutation feature importance and feature Shapley values were reported for models that predicted at least one true positive in the respective test data set. If a taxon was consistently ranked high in feature importance for a model according to permutation feature importance and/or SHAP value, then differential abundance of the taxon between the outcome groups was explored with the Wilcoxon rank-sum test.

See GitHub repository https://github.com/L-Coates/MILQ-Denmark for the commands used in QIIME2 to calculate alpha and beta diversities, and for the R code used to perform PERMANOVA, linear mixed effects modeling, differential abundance analysis, network analysis, logistic regression modeling, taxaHFE, and random forest modeling.

## RESULTS

### Sociodemographics, morbidity rates, and medicine/supplements frequencies by visit period

Infant gender, mode of birth (vaginal vs. cesarean section), mother’s parity, and household size were recorded. Among the subset of 109 infants analyzed, 93.6% were born vaginally, 73.4% were firstborn, and 65.1% lived in a three-member household (Table 1).

**TABLE 1:** Sociodemographic covariates of infants and their mothers

Sample set	Infant gender (*n*, percent of total dyads)	Mode of birth (*n*, percent of total dyads)	Mother’s parity (*n*, percent of total dyads)	Number people in household (*n*, percent of total dyads)
Infant stool 16S rRNA amplicon data (*n* = 327)	Male—47, 43.1% Female—62, 56.9%	Vaginal—102, 93.6% C-section—7, 6.4%	1–80, 73.4% 2–24, 22.0% 3–5, 4.6%	2–1, 0.9% 3–71, 65.1% 4–28, 25.7% 5–8, 7.3% 6–1, 0.9%

**Fig 1:**
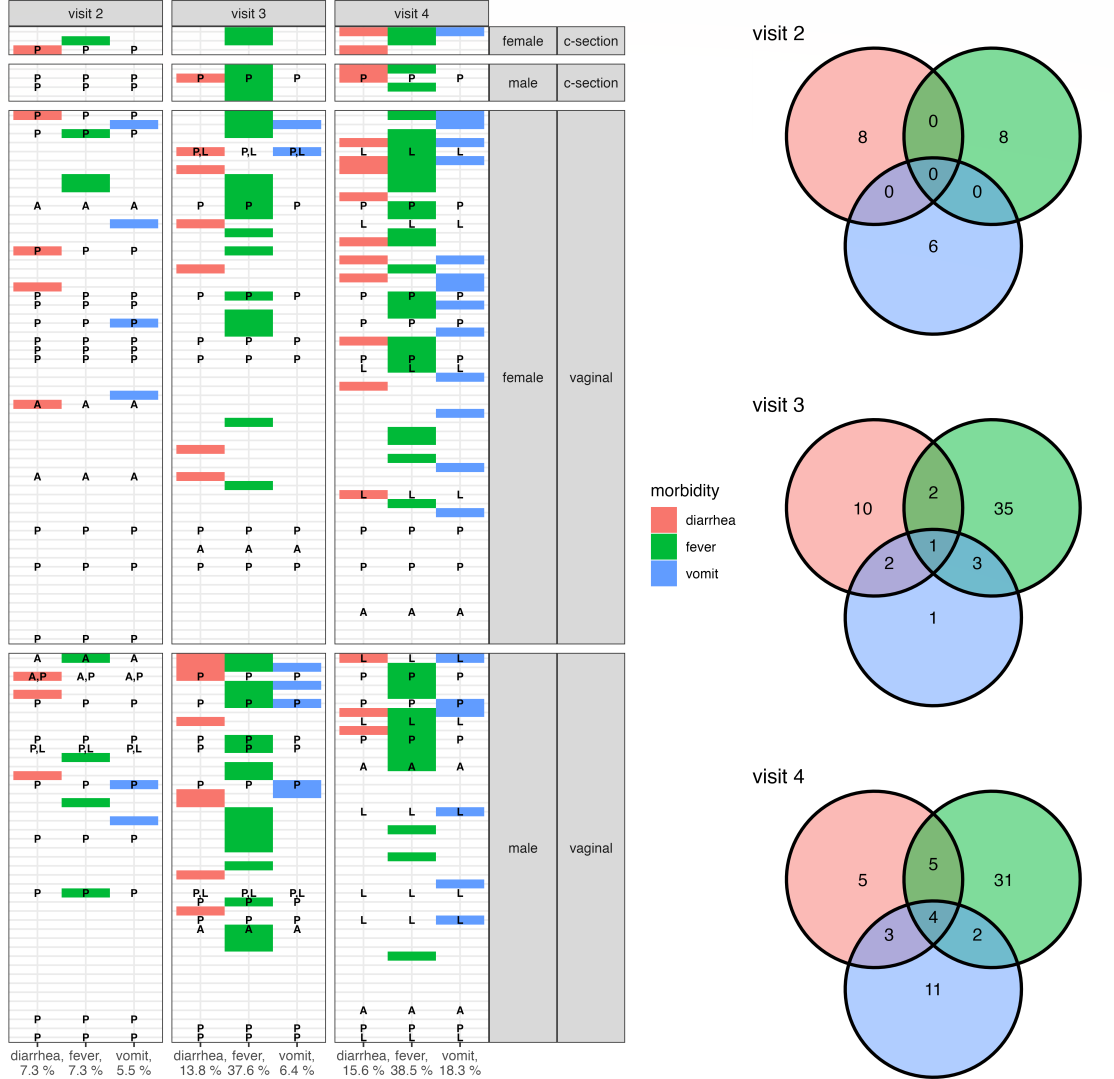
Color-coded list of morbidities, medications, and supplements for each infant within each visit period and Venn diagrams displaying the number of infants with one or more types of morbidity in a visit period. Gender and mode of birth are shown for each infant, and infants are listed in descending order of the total number of diarrhea, fever, and vomiting events during the study. Antibiotic (“A”), laxative (“L”), and probiotic (“P”) use during each visit period are shown for each infant.

For each visit, mothers reported occurrences of diarrhea, fever, and vomiting among their children. Out of 109 children, 86 (~79%) had at least one of these morbidities at one time from birth to 8.5 months of age (Fig. 1). Fever was the most common morbidity, and vomiting was the least common within each visit period. The per-visit prevalence of each morbidity displayed a pattern of increasing from visit 2 to visit 4, with diarrhea occurring in 15.6% of infants, fever occurring in 38.5% of infants, and vomiting occurring in 18.3% of infants by visit 4. Notably too, infants often did not have more than one of the morbidities of interest (diarrhea, fever, and vomit) within a visit period (Fig. 1). In visit 2, none of the infants had more than one morbidity type, 14.8% of the infants reported with morbidity in visit 3 had more than one morbidity type, and 23.0% of infants reported with morbidity in visit 4 had more than one morbidity type. Fever can frequently occur because of vaccination. All 109 infants in this analysis had vaccination reported for at least one visit period, usually for visits 3 or 4 (Fig. S3). However, fever events did not usually occur in the same visit period that a vaccination was recorded (i.e., <50% of infants with a vaccination had a fever in the same visit period), and infants that were not vaccinated in a particular visit period sometimes had fever in the same visit period (i.e., 0%–37% of infants without vaccination had a fever in the same visit period) (Fig. S3). Exact dates of morbidities and morbidity diagnoses were not gathered as part of the analyzed questionnaires. Rather, mothers were asked, for each morbidity separately, if the morbidity occurred within the week prior to the visit and if the morbidity occurred since the previous visit (excluding the week prior to the visit). Therefore, fever cases could not be linked to vaccinations, and morbidities that occurred in the same visit period for an infant cannot be identified as comorbidities.

Most infants (59.6%) experienced a fever at least once during the study, while less than half the infants experienced diarrhea (32.1%) or vomiting (25.7%) during the study (Table 2). If an infant had a morbidity during the study, it usually only occurred once and was rarely officially diagnosed or treated (Table 2). Among the few infants who were treated, mothers reported using antibiotics or acetaminophen to treat fever and antiemetics or painkillers to treat vomiting. Diarrheal symptoms usually lasted longer than fever or vomiting (median of 5.0, 1.8, and 1.0 days, respectively).

**TABLE 2:** Summary statistics of morbidity (diarrhea, fever, or vomit) cases across the subset of 109 infants analyzed^*a*^

Morbidity	Infants afflicted (*n*, percent of total infants)	Total cases	Cases per infant (min, median, max)	Duration, days (min, median, max)	Diagnosed cases (*n*, percent of total cases)	Treated cases (*n*, percent of total cases)
Diarrhea	35, 32.1%	42	1, 1, 2	1.0, 5.0, 25.0	6, 14.3%	0, 0%
Fever	65, 59.6%	105	1, 1, 4	1.0, 1.8, 8.5	22, 21.0%	8, 7.6%
Vomit	28, 25.7%	44	1, 1, 5	0.5, 1.0, 2.0	4, 9.1%	2, 4.5%

^*a*^ Number of infants afflicted with a morbidity, total morbidity cases, duration of cases, total diagnosed cases, and total treated cases.

Although infants were generally healthy (without known disease), some did receive antibiotics, probiotics, and laxatives (Fig. 1). Probiotic use was most common, followed by laxative use, and lastly, antibiotic use. The number of infants that were given probiotics was greatest during visit 2 (*n* = 24 in visit 2). Likewise, the number of infants that were given antibiotics was greatest in visit 2 (*n* = 5 in visit 2). The number of infants that were given laxatives was greatest during visit 4 (*n* = 10 in visit 4).

### Dominant microbes in infant stool

There were no taxa that were detected in all 327 stool samples at the ASV, species, genus, or family level, although *Streptococcus* and Streptococcaceae were detected in 325 stool samples. There were 11 genera and 11 families detected in more than 50% of the infant stool samples. These 11 genera included, in order of decreasing prevalence, with prevalence and median relative abundance (of rarefied counts) listed: *Streptococcus* (99.4%, 3.5%), *Veillonella* (89.0%, 2.6%), *Bifidobacterium* (83.5%, 25.4%), *Escherichia*/*Shigella* (71.3%, 2.6%), *Staphylococcus* (70.6%, 0.06%), *Actinomyces* (70.0%, 0.04%), *Enterococcus* (69.1%, 0.3%), *Clostridium sensu stricto 1* (65.1 %, 0.3%), *Lactobacillus* (63.6%, 0.2%), unknown Enterobacteriaceae genus (56.3%, 0.07%), and *Bacteroides* (56.3%, 1.3%). The 11 families included, in order of decreasing prevalence, with prevalence and median relative abundance (of rarefied counts) listed: Streptococcaceae (99.4%, 3.5%), Enterobacteriaceae (92.7%, 4.9%), Veillonellaceae (91.7%, 2.7%), Bifidobacteriaceae (84.7%, 25.4%), Actinomycetaceae (72.2%, 0.06%), Staphylococcaceae (70.6%, 0.06%), Enterococcaceae (69.1%, 0.3%), Clostridiaceae (65.4%, 0.3%), Lactobacillaceae (63.6%, 0.2%), Bacteroidaceae (56.3%, 1.3%), and Lachnospiraceae (54.7%, 0.03%).

### Infant characteristics and environmental factors associated with variation in stool microbial communities

First, batch effects from DNA extraction, PCR, and sequencing were evaluated for impact on DNA concentration, read depth, or microbial community structure. There was a difference in read depth by DNA extraction batch and PCR, but there was not a difference in DNA concentration by DNA extraction batch, and there was no difference in microbial community structure (measured by unweighted and weighted UniFrac distances) by DNA extraction, PCR, or sequencing batches. For those reasons, these variables were not included in the models presented hereafter.

Infant stool alpha diversity metrics—Shannon entropy (richness and richness), Faith’s phylogenetic diversity (phylogenetic relatedness/distances among taxa), total observed ASVs (number of taxa; blind to abundance), and Pielou’s evenness (similarity of species abundances)—were analyzed in relation to infant characteristics (age, gender) and environmental factors (antibiotic and supplement use, exclusive breastfeeding in first 4 months of life, mode of delivery, mother’s parity, household size) using linear mixed-effects modeling. Infant age was positively associated with Shannon entropy, Faith’s phylogenetic diversity, total observed ASVs, and Pielou’s evenness (*P*-values < 0.0001; Table 3; Fig. S4).

**TABLE 3:** Significant predictors of infant stool alpha diversity^*a*^

Response variable	Significant predictor variables	Beta coefficient	Standard error	*P*-value
Shannon entropy	Age (days)	5.100 × 10^−3^	6.455 × 10^−4^	7.33 × 10^−14^
	Antibiotic (yes)	−4.653 × 10^−1^	2.354 × 10^−1^	0.0490
	Diarrhea (yes)	−2.872 × 10^−1^	1.278 × 10^−1^	0.0253
Faith’s phylogenetic diversity	Age (days)	5.475 × 10^−3^	0.001152	3.29 × 10^−6^
	Gender (female)	0.389275	0.186228	0.0391
	Exclusive breastfeeding first 4 months (yes)	0.448509	0.220178	0.0441
Total observed ASVs	Age (days)	7.275 × 10^−3^	7.176 × 10^−4^	<2 × 10^−16^
	Antibiotic (yes)	−5.592 × 10^−1^	2.639 × 10^−1^	0.03497
Pielou’s evenness	Age (days)	5.448 × 10^−4^	1.038 × 10^−4^	3.09 × 10^−7^
	Household size (>3)	5.831 × 10^−2^	2.880 × 10^−2^	0.0455
	Diarrhea (yes)	−4.004 × 10^−2^	2.031 × 10^−2^	0.0496

^*a*^ The beta coefficient and associated standard error and *P*-value are from the full models predicting alpha diversity.

Infant females had greater Faith’s phylogenetic diversity than infant males (*P*-value < 0.05; Table 3), and infants exclusively breastfed the first 4 months of life had greater Faith’s phylogenetic diversity than those not exclusively breastfed (*P*-value < 0.05; Table 3). Infant antibiotic use was negatively associated with Shannon entropy and total observed ASVs (*P*-values < 0.05; Table 3). Infants living in households with more than three individuals had greater Pielou’s evenness in their stool than those living in households with three or fewer individuals (*P*-value < 0.05; Table 3).

The microbial community structure in infant stool samples was further explored by calculating weighted and unweighted UniFrac distances (beta diversity) between all samples and ordinating with principal coordinates analysis (Fig. S5). Subsequently, the ASVs that contributed the most to variance were determined using a covariance matrix calculated within the QIIME2 wrapper for PCoA biplot. For unweighted UniFrac distances, two ASVs contributed largely to separation among samples—one belonging to *Bifidobacterium* and one belonging to *Bacteroides* (Fig. S5A). Notably, the *Bifidobacterium* ASV was associated with the clustering of samples along Axis 2. These two ASVs, along with an ASV belonging to Clostridium *sensu stricto* 1, contributed notably to separation among samples by weighted UniFrac distances (Fig. S5B). When employing PERMANOVA, none of the covariates were found to significantly associate with UniFrac distances among infant stool microbiota. Furthermore, stool samples that clustered along Axis 2 (and tended to contain the *Bifidobacterium* ASV, irrespective of abundance) in the PCoA of unweighted UniFrac distances (Fig. S5A) did not tend to come from infants that received probiotics, as evidenced by similar proportions of stool samples from infants taking probiotics in both clusters (i.e., 16.7% in the *Bifidobacterium* ASV-associated cluster versus in 16.2% in the larger cluster). Stool samples in this cluster had greater Faith’s phylogenetic diversity (mean 10.4 ± 1.29 SD) than the other stool samples (mean 6.36 ± 1.25 SD) by a Wilcoxon rank-sum test (*P*-value < 0.05).

Weighted and unweighted UniFrac distances were also calculated within each visit separately (i.e., cross-sectional data) and related to covariates using PERMANOVA. Infant antibiotic use in visit 2 was associated with unweighted UniFrac distances (*P*-value = 0.020; Fig. S6A), and gender was associated with weighted UniFrac distances in visit 2 (*P*-value = 0.041; Fig. S6B), and exclusive breastfeeding before 4 months was associated with unweighted UniFrac distances in visit 4 (*P*-value = 0.004; Fig. S6C).

ANCOM-BC2 was used to determine infant stool taxa differentially abundant with covariates. Infant age was associated with an abundance of 15 different stool microbial genera (Fig. S7). Most of these genera increased over the course of 0.8–8.5 months of age, and this included *Enterococcus*, *Veillonella*, Escherichia-Shigella, (Ruminococcus) gnavus group, Clostridium *sensu stricto* 1, *Bifidobacterium*, *Clostridioides*, *Granulicatella*, and *Lachnoclostridium*. Only two stool bacterial genera decreased over 0.8–8.5 months of age: *Corynebacterium* and *Staphylococcus*.

Infant probiotic use was positively associated with stool *Lactobacillus rhamnosus* abundance according to ANCOM-BC2 (FDR-adjusted *P*-value = 0.049) and Wilcoxon rank-sum test (*P*-value = 0.0002; Fig. S8). Thirteen out of 24 of these infants that received probiotics were given Lactocare Baby probiotic (Karo Healthcare AB, Sweden), which contains *L. rhamnosus* and *Lactobacillus reuteri*. Meanwhile, five other infants were given DUOLAC probiotic (containing bifidobacteria), three infants were given BioGaia products (containing *L. reuteri*), and two infants’ mothers did not report the probiotic product name.

### Infant stool microbial associations with morbidity

Since the exact dates of infant morbidity were not recorded, the days elapsed between morbidity and stool collection cannot be determined. Stool collection definitely occurred within the same week (i.e., within 0–6 days) of morbidity for infants whose mothers reported that morbidity and stool collection both occurred within the week prior to the morbidity interview. Although there were 298/327 infant stool samples collected within the week prior to the morbidity interview (Fig. S9), relatively few infants had a morbidity during the same week of the stool collection and morbidity interview: 17.5%, 27.5%, and 18.2% of diarrhea-, fever-, and vomit-afflicted infants, respectively, had stool collected within the same week as the morbidity (Table 4). More often, stool was collected after the morbidity reported for that visit, within an unknown number of days elapsed between them: 77.5%, 63.7%, and 66.7% of diarrhea-, fever-, and vomit-afflicted infants had a stool sample collected after the morbidity. Infrequently, stool was collected before the morbidity reported for that visit period: 2.5%, 3.3%, and 0.0% for diarrhea-, fever-, and vomit-afflicted infants. And for 2.5%, 5.5%, and 15.2% of diarrhea-, fever-, and vomit-afflicted infants, the relative timing is unknown for stool collection to morbidity reported for that visit. Consequently, for most morbidity-afflicted infants, it is unknown whether their stool samples were collected near the time of morbidity. On the other hand, it is known that most (>63%) of stool samples were collected after diarrhea, fever, or vomiting had at least begun, providing context to relationships identified between these morbidities and the stool microbiota.

**TABLE 4:** Relative timing of infant morbidity to stool collection within the same visit^*a*^

Morbidity (*n* = total corresponding stool samples)	Stool collection and morbidity reported in the same week (*n*, percent)	Stool collected before morbidity (*n*, percent)	Stool collected after morbidity (*n*, percent)	Infants with unknown relative timing of morbidity to stool collection (*n*, percent)
Diarrhea (*n* = 40)	7, 17.5%	1, 2.5%	31, 77.5%	1, 2.5%
Fever (*n* = 91)	25, 27.5%	3, 3.3%	58, 63.7%	5, 5.5%
Vomit (*n* = 33)	6, 18.2%	0	22, 66.7%	5, 15.2%

^*a*^ Number and percent of stool collected within the same week as morbidity, collected before morbidity, collected after morbidity, or collected at an unknown time relative to morbidity.

Shannon entropy and Pielou’s evenness were negatively associated with diarrhea (*P*-value < 0.05; Table 3). With a reduced model that only included age and antibiotic use as covariates, the total observed ASVs metric was negatively associated with diarrhea (*P*-value < 0.05). Neither diarrhea, fever, nor vomiting in any of the visit periods was associated with weighted or unweighted UniFrac distances.

**Fig 2:**
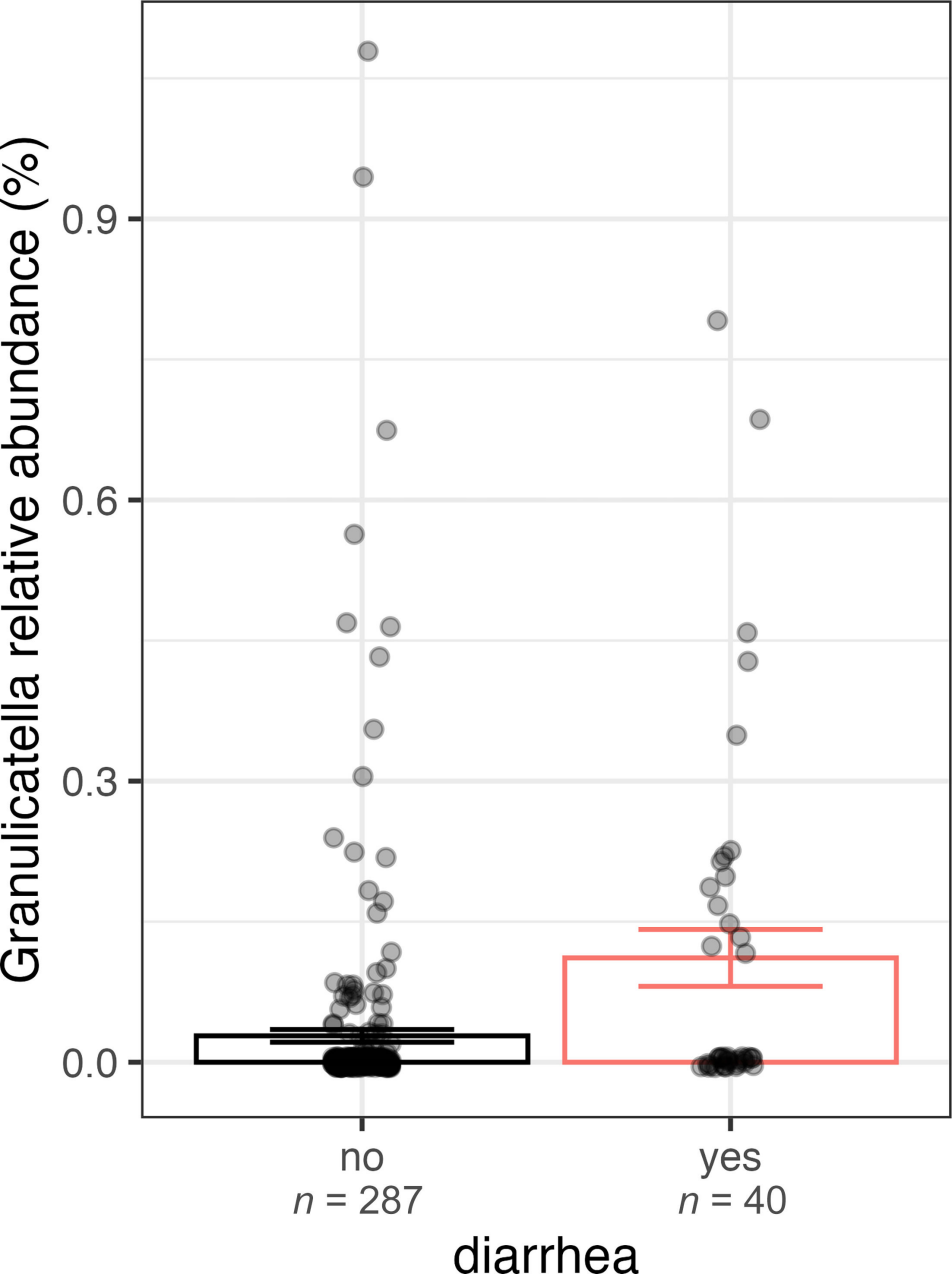
*Granulicatella* relative abundance (%, from rarefied counts) in infant stool collected when the infant had diarrhea in the same visit period, or did not have diarrhea in the same visit period. Mean and standard error of *Granulicatella* relative abundance are displayed for each group.

**Fig 3:**
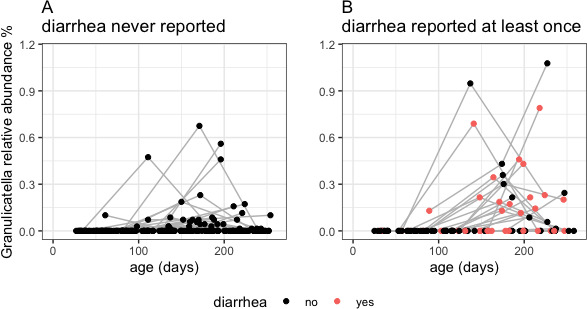
Scatter plot of *Granulicatella* relative abundance (%, from rarefied counts) in stool across all three visits for infants that never had diarrhea in the study (**A**) or had diarrhea at least once in the study (**B**).

**Fig 4:**
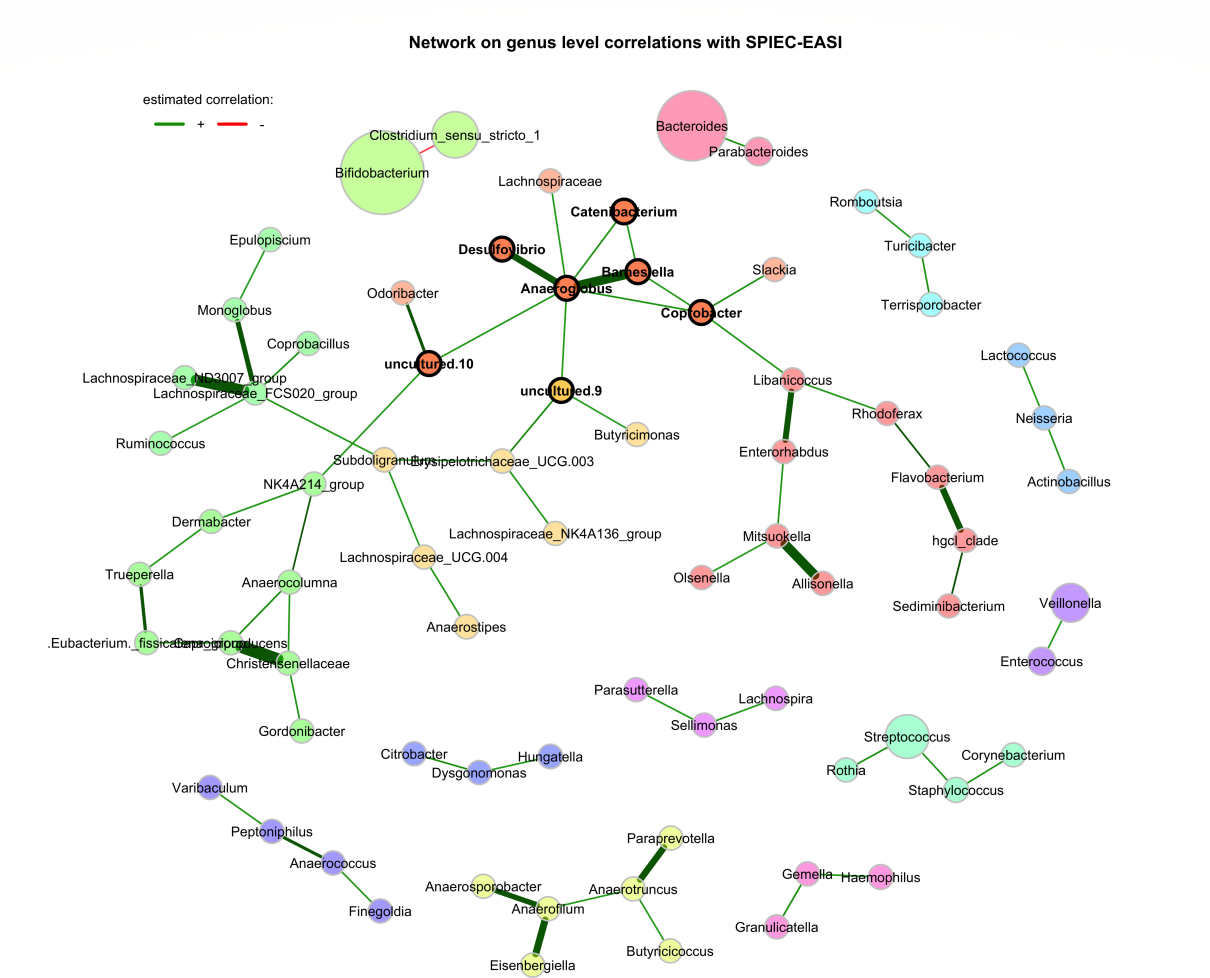
Network of infant stool microbes determined with NetCoMi using the SPIEC-EASI method for microbial correlation. Positive correlations are depicted with green edges, and negative correlations are depicted with red edges. Microbial genera are depicted as nodes, and the node size is proportional to the sum of the normalized abundance of the genus. The edge weight is proportional to the strength of the connection (estimated correlation) between nodes (microbial genera).

Nonetheless, a positive association was found between *Granulicatella* abundance in the stool and infant diarrhea in the same visit period, when using ANCOM-BC2 (log fold change: 1.07, adjusted *P*-value = 0.029 for full model) and secondarily, one-sided Wilcoxon rank-sum test with counts rarefied at 6,963 without replacement (Fig. 2; *P*-value < 0.0001). A pattern was observed: *Granulicatella* did not appear in infant stool until about 100 days of age (with the exception of three infant stool samples), in both infants that had diarrhea at least once (Fig. 3A) and infants that never had diarrhea (Fig. 3B). After 100 days of age was when most infants (82 out of 109) were first given a complementary fluid (including water) or food (Fig. S10). Of the 27 infants given complementary fluid/food before 100 days of age, 20 were introduced to complementary fluid/food within the first week of birth, and in most cases, it was infant formula. However, within stool samples from the same infant, *Granulicatella* did not display a pattern of appearance or increasing abundance soon after introduction of complementary fluids/foods (Fig. S11A). Likewise, infants with >0.00% relative abundance of *Granulicatella* in at least one stool sample were not introduced to complementary fluids/foods earlier than infants that had 0.00% relative abundance of *Granulicatella* in all stools (Fig. S11B). Next, correlations among infant stool microbes were determined using SPIEC-EASI and visualized with NetCoMi (Fig. 4). *Granulicatella* was positively correlated with *Gemella*.

**Fig 5:**
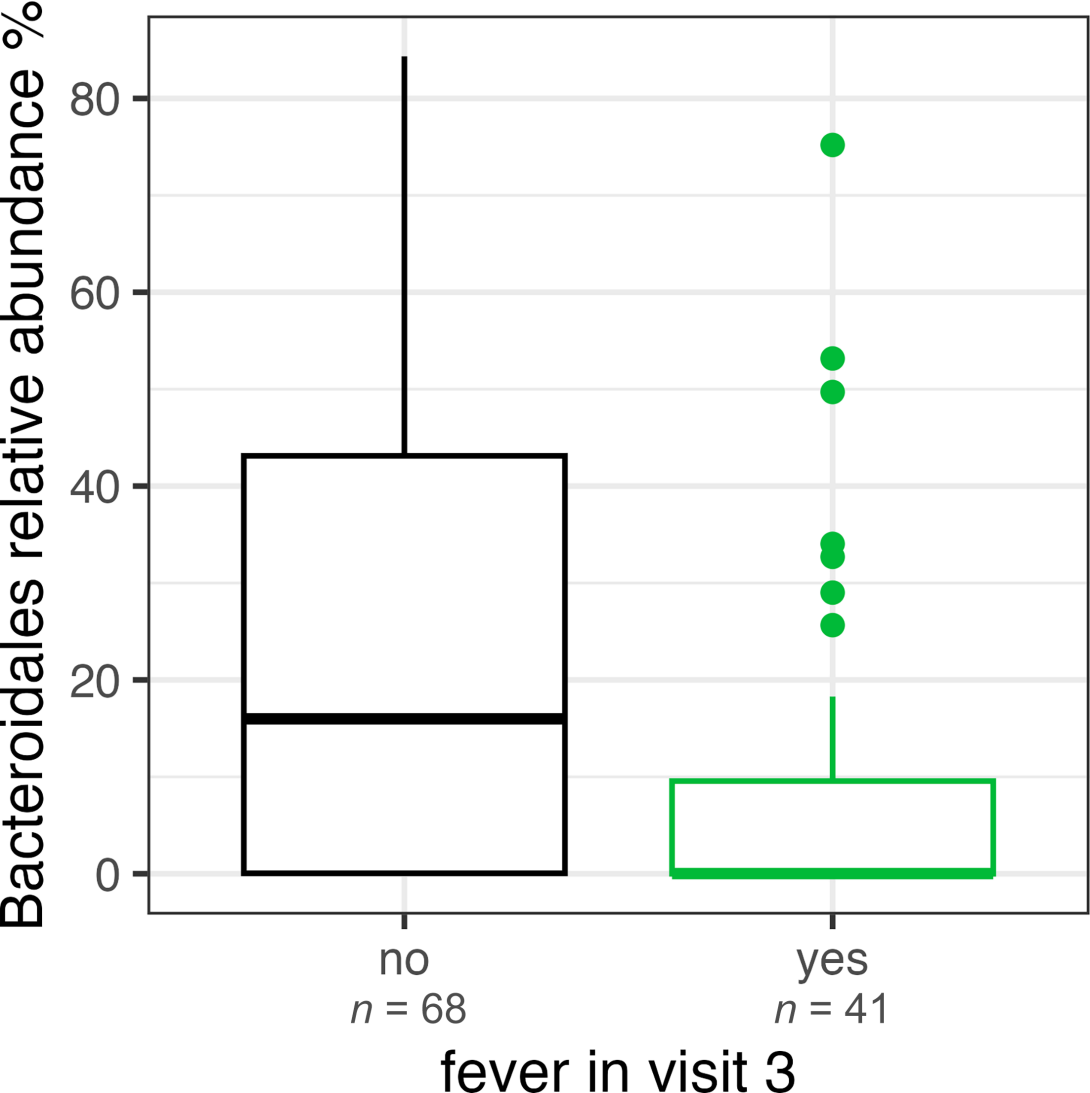
Box plot of *Bacteroidales* relative abundance (%, from rarefied counts) in visit 3 infant stool collected when the infant had fever in the same visit period, or did not have fever in the same visit period.

Associations between stool microbial abundance and morbidity were also investigated within each of the three visits, separately (i.e., three separate cross-sectional analyses). In visit 3, stool Bacteroidales abundance was negatively associated with fever by ANCOM-BC2 (FDR-adjusted *P*-value = 0.016) when using a reduced model that included age and antibiotic use (which were the only two covariates that significantly associated with a microbial taxa at the order level). Follow-up Wilcoxon rank-sum test also confirmed an association between fever in visit 3 and reduced Bacteroidales abundance (*P*-value = 0.0001; Fig. 5). In visit 3 samples, *Bacteroides*, *Parabacteroides,* and *Butyricimonas* were the three most abundant genera, on average, belonging to Bacteroidales. No associations were found between stool microbial abundance and morbidity at visit 2 or visit 4.

### Linking early gut microbiome to morbidity later in infancy

**Fig 6:**
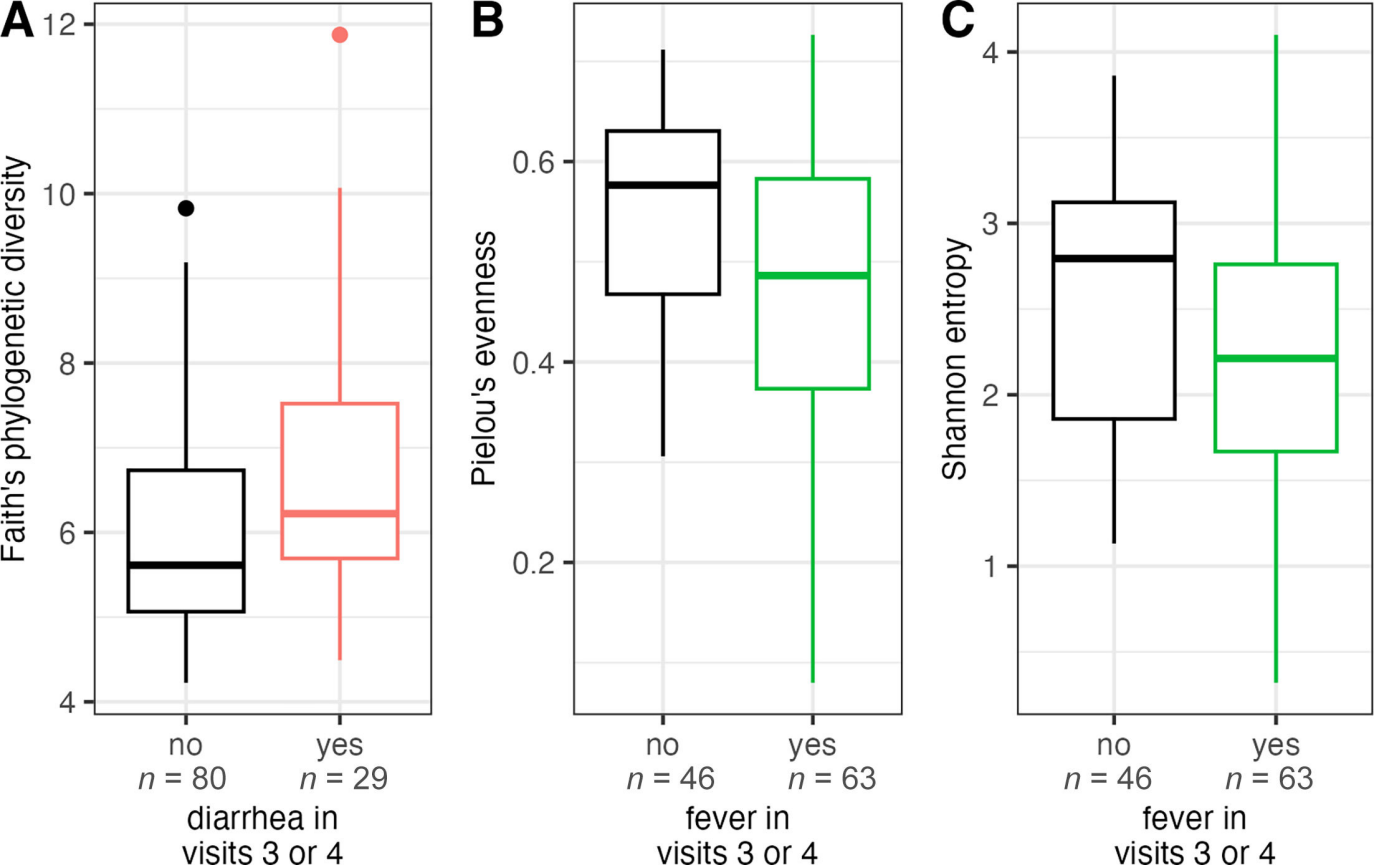
Box plot of Faith’s phylogenetic diversity (**A**), Pielou’s evenness (**B**), and Shannon entropy (**C**) in visit 2 collected from infants that developed or did not develop diarrhea (**A**) or fever (**B**) in visits 3 or 4.

To determine whether early gut microbiota can predict later occurrence of diarrhea, fever, or vomiting, microbiota from stool samples collected at visit 2 was analyzed relative to morbidity outcomes during visits 3 and/or 4. The unweighted and weighted UniFrac distances of stool microbiota at visit 2 were not associated with diarrhea, fever, or vomiting at visits 3 and/or 4 as determined by PERMANOVA. Furthermore, logistic regression was employed to determine if alpha diversity during visit 2 predicted one of the morbidity outcomes in visits 3 and/or 4. Faith’s phylogenetic diversity in visit 2 was positively associated with diarrhea in visits 3 and/or 4 (Table 5). And, according to the follow-up one-sided Wilcoxon rank-sum test and one-sided t-test (on box-cox transformed values), Faith’s phylogenetic diversity in visit 2 was also greater among infants that had diarrhea in visits 3 or 4 versus those that did not (*P*-value < 0.05; Fig. 6A). Pielou’s evenness in visit 2 was negatively associated with fever in visits 3 or 4 (Table 5), and subsequent one-sided Wilcoxon rank-sum test indicated that Pielou’s evenness in visit 2 was greater among infants that did not have fever in visits 3 or 4 versus those that did (*P*-value < 0.005; Fig. 6B). Additionally, Shannon entropy was negatively associated with fever at visits 3 and 4 (Table 5), and a follow-up one-sided t-test indicated that Shannon entropy was greater among infants that did not develop fever in visits 3 or 4 versus infants that did (*P*-value < 0.05; Fig. 6C). All three models did poorly in predicting diarrhea or fever outcome in visits 3 and 4, as evidenced by a low balanced accuracy no better than chance, low F1-score, and low ROC AUC.

**TABLE 5:** Logistic regression model performance predicting morbidity in visits 3 and 4 using alpha diversity at visit 2^*a*^

Morbidity outcome in visits 3 or 4	Alpha-diversity metric	Odds ratio	*P*-value	Balanced accuracy	F1-score	ROC AUC
Diarrhea	Faith’s phylogenetic diversity	1.62	0.0227	0.500	0.167	0.526
Fever	Pielou’s evenness	0.000770	0.00408	0.459	0.579	0.526
Fever	Shannon entropy	0.418	0.0211	0.459	0.579	0.496

^*a*^ Listed are models for which the alpha-diversity metric in visit 2 had a significant association with morbidity in visits 3 and 4 using the training data set. Models also included as covariates morbidity in visit 2 and the number of people in the household.

Abundances of stool microbes in visit 2 were then explored as potential predictors of morbidity outcomes in visits 3 and 4. First, taxaHFE version 2.0 (39) was utilized with the rarefied counts to select stool microbial taxa from visit 2 that could be predictive of morbidity outcome in visits 3 and 4. These taxa were included in the random forest model to predict the respective morbidity outcome (Table 6).

**TABLE 6:** Early microbial predictors of later morbidity^*a*^

Morbidity outcome in visit 3 or 4	Microbial abundance in visit 2 (taxa selected with taxaHFE)	Balanced accuracy	F1-score	ROC AUC
Diarrhea	*Bacteroides vulgatus*, *Bacteroides uniformis*, Corynebacteriales, Flavobacteriales, *Haemophilus*, *Prevotella timonensis*, *Rhodoferax*, *Sutterella wadsworthensis*, and Staphylococcales	0.646	0.462	0.615
fever	Actinobacteriota, Bacilli, *Bacteroides fragilis*, *Negativicutes*, and unknown human gut species of Clostridium *sensu stricto* 1	0.603	0.667	0.620
Vomit	*Actinomyces, Bacteroides caccae*, *Bacteroides thetaiotaomicron, Bifidobacterium bifidum*, *Bifidobacterium dentium*, *Clostridium butyricum*, *Corynebacterium pseudodiphtheriticum*, *Erysipelatoclostridium ramosum*, Flavobacteriales, Gemellaceae, *Haemophilus*, *Lactobacillus reuteri*, *Veillonella ratti*, *Parabacteroides distasonis*, *Raoultella*, *Streptococcus agalactiae*, and uncultured Bacteroidetes (of *Sediminibacterium*)	0.480	0 (due to 0 true positives)	0.857

^*a*^ Statistical results and performance of random forest models are displayed. For each model, household size, morbidity in visit 2, and microbial abundances in visit 2 were predictor variables, and morbidity in visits 3 and/or 4 as was the response variable.

**Fig 7:**
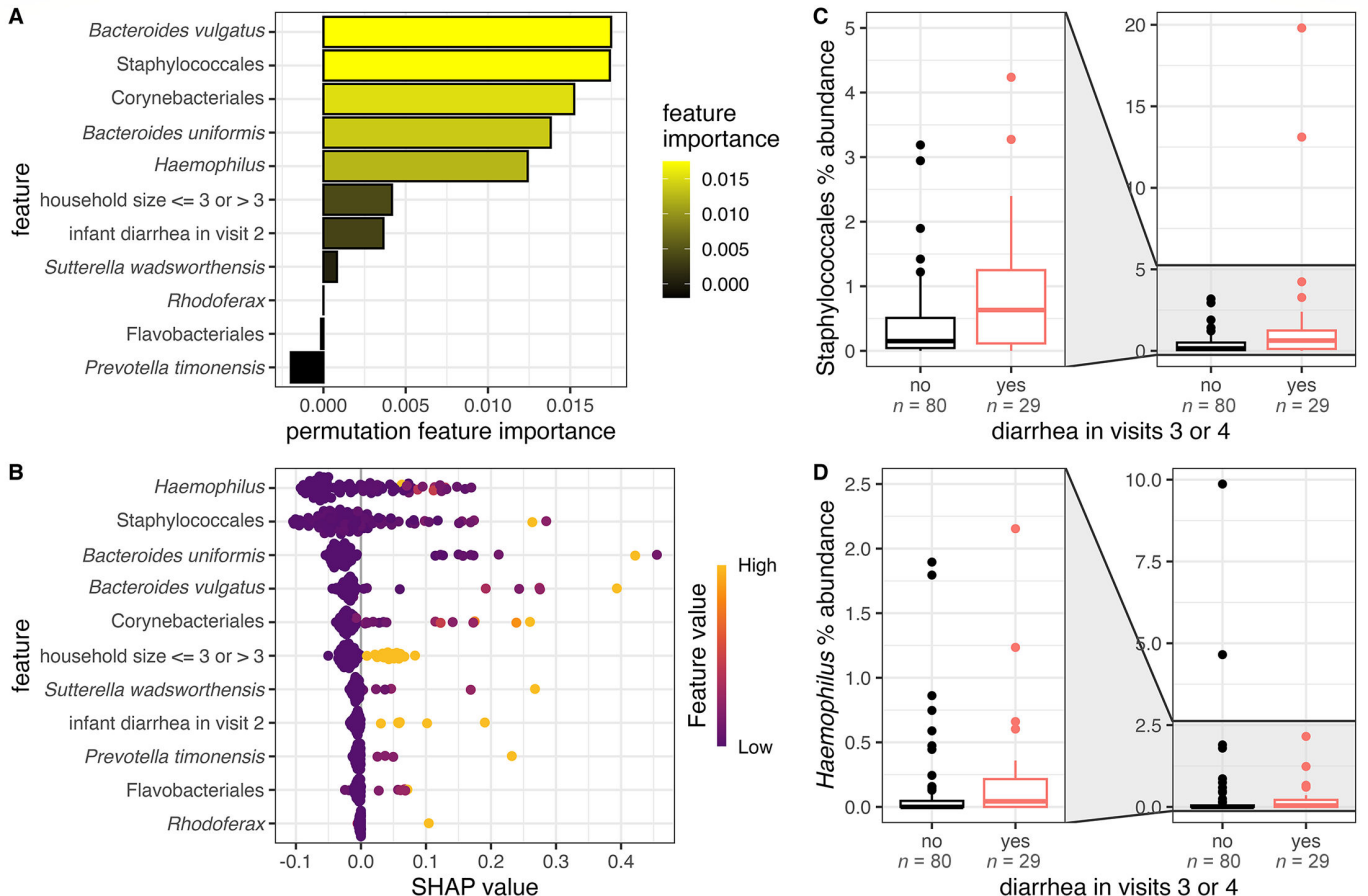
Importance of features in the random forest model predicting diarrhea outcome in visits 3 or 4. The bar graph of permutation feature importance (**A**), and the dot plot of feature SHAP values (**B**) indicate Staphylococcales (**C**) and *Haemophilus* relative abundances in visit 2 (**D**) as important features in predicting diarrhea outcome in visits 3 or 4.

Infant morbidity in visit 2 and household size were also included in the models. The random forest model for diarrhea outcome (in visits 3 and 4) had a balanced accuracy and ROC AUC above 0.6, but the F1-score was low due to high false negatives (recall was 0.375) (Table 6). Among the features used for the random forest model of diarrhea outcome in visits 3 and 4, *Bacteroides vulgatus* and Staphylococcales relative abundances were high in permutation feature importance to the model (Fig. 7A) while SHAP values indicated that low *Haemophilus* and Staphylococcales relative abundances were usually important in predicting no diarrhea in the test data (Fig. 7B). Post hoc one-sided Wilcoxon rank-sum tests were performed for *B. vulgatus*, Staphylococcales, and *Haemophilus* relative abundances (from rarefied counts) by diarrhea outcome in visits 3 or 4. Staphylococcales and *Haemophilus* were significantly greater in visit 2 samples from infants who reported diarrhea at visits 3 or 4 versus infants who had not (adjusted *P*-values < 0.005 and < 0.05, respectively; Fig. 7C and D). Among the stool samples collected at visit 2, Staphylococcales was only represented by the genera *Staphylococcus* and *Gemella*.

**Fig 8:**
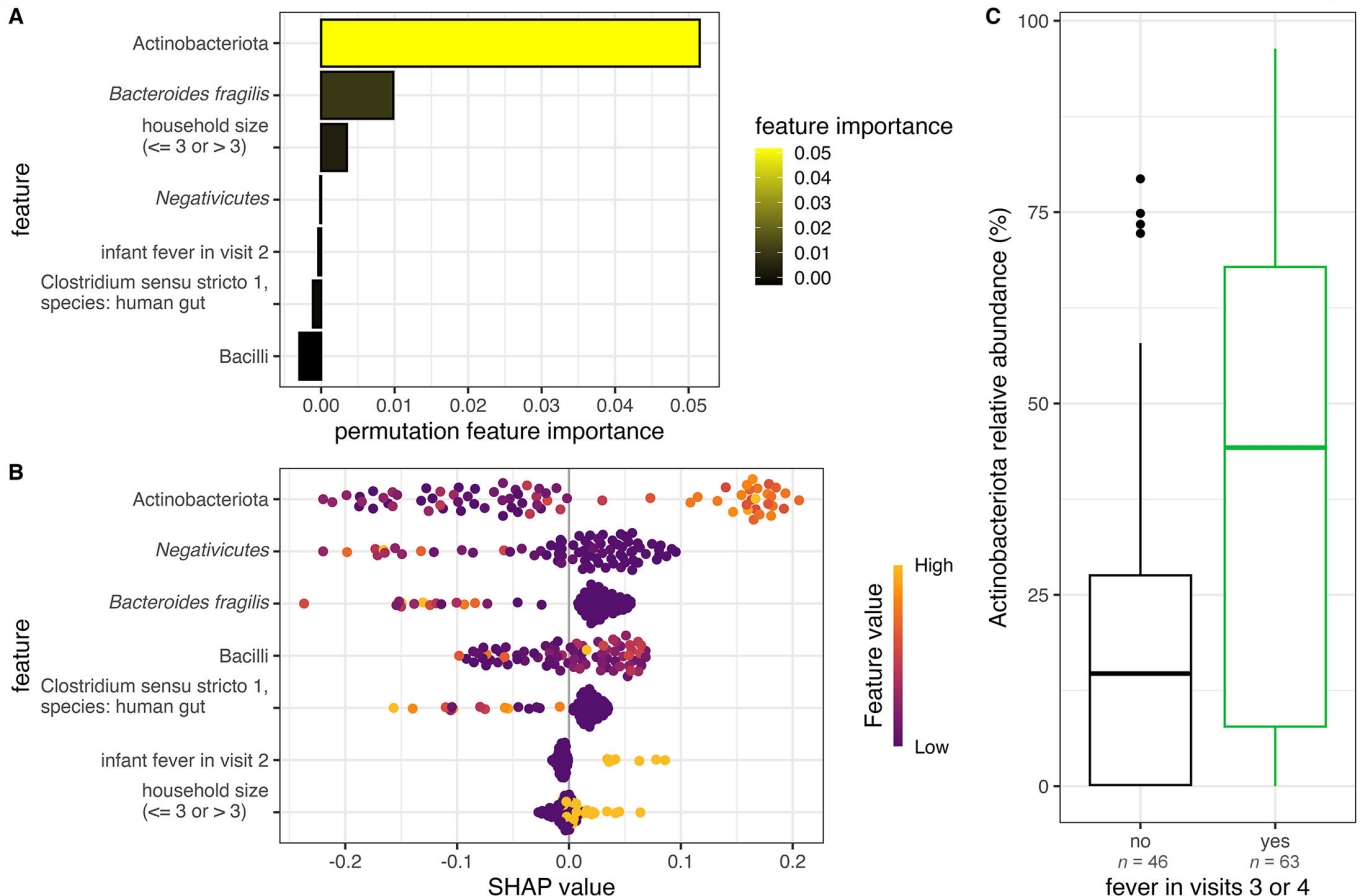
Importance of features in the random forest model predicting fever outcome in visits 3 or 4. The bar graph of permutation feature importance (**A**), and the dot plot of feature SHAP values (**B**), both indicate Actinobacteriota relative abundance in visit 2 (**C**) as an important feature in predicting fever outcome in visits 3 or 4.

For the random forest model of fever outcome in visits 3 and 4, Actinobacteriota relative abundance was the highest in both permutation feature importance (Fig. 8A) and SHAP value (Fig. 8B) among the model features. Also, high Actinobacteriota abundance was usually important in predicting fever for visits 3 and 4 (Fig. 8B). Actinobacteriota relative abundance (from rarefied counts) was also significantly greater at visit 2 from infants who reported fever at visits 3 or 4 versus infants who had not (*P*-value < 0.005; Fig. 8C). Actinobacteroita detected in stool samples at visit 2 were comprised primarily of *Bifidobacterium*, *Collinsella*, and *Actinomyces*.

## DISCUSSION

As far as the authors are aware, this study is the first to explore the associations between gut bacterial taxa and gastroenteritis-associated morbidities (diarrhea, fever, and vomiting) among breastfeeding Danish infants during the first 8.5 months of life. In light of observations that infant gut microbiomes vary with geographical location, ethnicity, and breastfeeding (1), and that diarrhea etiologies occur at different rates according to geographical region (14), studying this Danish breastfeeding infant cohort is helpful in developing a more comprehensive understanding of the causes and impacts of infant gastroenteritis. Several findings from this study corroborate past reports and, in doing so, justify, to some extent, this study design. For instance, findings reported here sustain previously published findings of a positive association between infant age and alpha diversity (1), a negative association between antibiotic use and stool alpha diversity (40), a negative association between diarrhea and stool alpha diversity (25, 41), and a positive association between household size and Pielou’s evenness (42). Antibiotic use was associated with Shannon entropy and total observed ASVs metrics and was not associated with phylogenetic diversity, indicating that the antibiotics used among this infant cohort may have had a broad impact among gut microbes. While diarrhea was associated with Shannon entropy and Pielou’s evenness, it was not associated with total observed ASVs or Phylogenetic diversity, suggesting that diarrhea may have altered the relative abundance of microbial taxa more so than which taxa were present in the gut microbial community. In a meta-analysis comparing exclusively breastfed to non-exclusively breastfed infants, exclusively breastfed infants had lower Faith’s phylogenetic diversity when evaluated across seven cohorts, including Bangladesh, Canada, Haiti, South Africa, and three cohorts in the United States (43). However, among the Danish cohort reported here, exclusive breastfeeding during the first 4 months of life was associated with slightly greater phylogenetic diversity in the infant gut compared to infants who received complementary foods during this time. This observation was similar to a subset of the cohorts contained in the meta-analysis, including Haiti and the United States, North Carolina (43), and might highlight potential for regionally specific environmental microbes to drive the relationship between gut microbial diversity and breast milk. Lastly, several gut microbial genera varied with age in this study, as they had varied with age in past studies of the infant gut microbiome. *Bifidobacterium*, *Erysipelatoclostridium*, *Escherichia-Shigella*, and *Lachnoclostridium* increased with age, and *Staphylococcus* decreased with age (44, 45).

This study found several relationships between the infant gut microbiome and morbidity, which help refine hypotheses about how gut microbes may contribute to or respond to diarrhea or fever. Only one taxon in the stool*—Granulicatella*—was found to associate with recent diarrhea (i.e., from the same visit period) in this cohort. *Granulicatella* is not a known etiological agent of diarrhea, and yet it has been positively associated with diarrhea among children in Malawi (22) and other low-income countries (25).

*Granulicatella* is a genus of catalase-negative facultative anaerobes belonging to the family *Carnobacteriaceae* and containing three known species*—G. adiacens*, *G. balaenopterae*, and *G. elegans* (46). *G. adiacens* and *G. elegans* have both been detected in oral samples of infants (47) as well as adults, and in breast milk (48–50). These bacterial species are infrequently identified as opportunistic pathogens, causative agents of endodontic infection (51), and endocarditis (inflammation of the inner lining of the heart chambers and valves) (46, 52). An analysis of the secretome of *G. adiacens* found more than 20% of the proteins were putative virulence proteins, and among them were superoxide dismutase and serine proteases (53). Superoxide dismutase, along with glutathione peroxidase, which is encoded in the genome as well, may enable *G. adiacens* to survive oxidative stress. *G. elegans* bacteremia has also been reported in association with abdominal diseases requiring operation (54). Although *Granulicatella* has been detected in human stool samples and there is strong evidence that it may act as an opportunistic pathogen, whether it colonizes the gastrointestinal tract or is a transient coincidental presence during disease remains unknown.

A longitudinal study of stool samples collected from children and adults with diarrhea caused by *Vibrio cholerae* or enterotoxigenic *E. coli* diarrhea found that, for both etiological agents of diarrhea, there was a pattern of microbial succession during recovery (27). The earliest bacteria dominating stool microbial communities after diarrhea were facultative anaerobes. The authors hypothesized that this may be in part due to increased levels of oxygen in the lumen following infection. If not an etiological agent of diarrhea, *Granulicatella* may increase in abundance following diarrhea because of its ability to survive in this more oxygen-rich intestinal environment (27). The data presented here may better support this latter hypothesis because 77.5% of stool samples from diarrhea-afflicted infants were collected after diarrhea onset, suggesting that primarily the gut microbiome signal during recovery from diarrhea was detected.

*Granulicatella* stool abundance was also positively correlated with *Gemella* abundance in the stool. *Gemella* abundance in stool has previously been positively associated with diarrhea in Vietnamese children (55). *Gemella* is also a facultative anaerobe, a consistent colonizer of the infant oral cavity (56), and is detected in breast milk (57). It has been observed to increase in abundance with age in the oral cavity, albeit not quite as pronounced as *Granulicatella* (56), and decrease in abundance in the breast milk throughout lactation (50). Although in this study, *Gemella* abundance in the stool was not associated with age, the positive correlation between *Gemella* and *Granulicatella* suggests conditions that promote *Granulicatella* abundance in the stool may also promote *Gemella* abundance.

Random forest modeling in this cohort suggests that infant gut microbes in the first 3.5 months of life are minimally predictive of gastroenteritis during 3.5–8.5 months of age. Assessment of feature importance identified Staphylococcales and *Haemophilus* as important to the prediction of diarrhea. Greater abundances of these microbes tended to be present in the first 3.5 months of life for infants that later developed diarrhea. These Danish infants had *Staphylococcus* and *Gemella* as the only genera belonging to Staphylococcales. The species of *Staphylococcus* present in this cohort could not be identified. However, other studies have found *Staphylococcus aureus* in infant stool samples. Enterotoxin-producing *S. aureus* can cause vomiting and diarrhea and has been associated with allergies in children (58). *Haemophilus parainfluenzae* has been positively associated with inflammatory bowel disease among children (59). In juvenile rhesus macaques with idiopathic chronic diarrhea, *H. influenzae* was reported to express more fucose permease, while *Bacteroides* spp. expressed more of the alpha-L-fucosidase enzyme capable of cleaving fucose from mucin (60). It was hypothesized that the etiology of idiopathic chronic diarrhea involved, in part, stimulation of host production of fucosylated mucin by mucin-degrading commensals, *Bacteroides*, and subsequent cross-feeding of free fucose to pro-inflammatory *Haemophilus*. Interestingly, in this Danish cohort, abundances of *Haemophilus* and a couple of *Bacteroides* spp. were important features in the model predicting future diarrhea from stool microbes in the first few months of life. Perhaps inflammation induced by *Haemophilus* and sustained by cross-feeding from commensals can increase diarrhea risk in healthy infants.

Gut microbial associations with fever and vomiting were also investigated, as these can be symptoms of intestinal infection. Unexpectedly, we found that diarrhea, fever, and vomiting rarely occurred in the same visit period for an infant. This suggests that diarrhea, fever, and vomiting events usually arise either from separate infections of the same etiological agent or different etiological agents. This may partially explain why there was no similarity among the taxa associated with these morbidities. Fever was negatively associated with Bacteroidales abundance in visit 3, and vomiting was not associated with any taxa. The lower abundance of Bacteroidales among infants with fever in visit 3 may be consistent with a past report of decreased Bacteroidales abundance in infants with acute febrile illness (Kawasaki disease) relative to healthy infants (61). *Bacteroides* and *Parabacteroides* were the two most abundant genera of Bacteroidales in this Danish infant cohort and included the species *Bacteroides fragilis*, *B. thetaiotaomicron*, *Parabacteroides distasonis*, and *P. goldsteinii,* which can induce production of interleukin-10 (62, 63)—an anti-inflammatory and antipyretic cytokine (64). These Bacteroidales species may have provided some protection against fever in Danish infants, but why this relationship was only detected in visit 3 is not immediately apparent. Median relative abundance of Bacteroidales was greatest in visit 3. Interestingly too, all but two infants received a vaccine during the period immediately prior to visit 3—a vaccination rate much higher than those in visits 2 and 4. It is tempting to speculate that Bacteroidales in the infant gut may have protected against vaccine-induced fever; however, it is unknown if any of the fever events were a result of vaccination because fever diagnoses were not collected.

Surprisingly, Actinobacteriota abundance in the first 3.5 months of life was greater in infants who developed fever later. *Bifidobacterium* and *Collinsella* were the two most abundant Actinobacteriota genera in this cohort and are associated with infant health (65). *Bifidobacterium* has also been associated with improved immune function (66) and immune memory after vaccination (67). A study relating the pediatric gut microbiota to duration of fever post-allogeneic hematopoietic stem cell transplantation reported a positive association between *Collinsella* abundance prior to transplantation and febrile duration post-transplantation (68), suggesting this bacterium may contribute to an overreactive immune response to allogeneic hematopoietic stem cell transplantation. Actinomyces was the third most abundant Actinobacteriota genus in this cohort, and it is an intestinal pathobiont (69). Fever is often a symptom of microbial infection and is beneficial to limiting pathogen growth and replication (9). Among this Danish infant cohort, there were many fever events that did not last longer than 2 days or require treatment. These fever events may have represented effective immune responses to infections that were successfully cleared. One might speculate that high *Bifidobacterium* abundance in the first few months of life contributed to these successful immune responses to microbial infection later in infancy. On the other hand, there were some infants who had fever lasting several days or who were potentially severe enough to require treatment. These cases may have represented serious microbial infections or impaired immune function. One may hypothesize that a high abundance of *Collinsella* or *Actinomyces* early in life may contribute to these more severe fever events later in life.

In summary, the study presented here contributes to the understanding of the relationships of the gut microbiota with diarrhea and fever during early life, especially among well-nourished, breastfeeding infants. Diarrhea continues to be an important cause of morbidity in high-income countries, and in this Danish cohort, ~32% of infants experienced diarrhea at some time during the first 8.5 months of life. No bacterial etiological agents of diarrhea were associated with recent diarrhea, although Staphylococcales abundance within the first 3.5 months was associated with diarrhea after 3.5 months, which may indicate the Danish infants were often infected with viral etiological agents of diarrhea. In fact, a previous study of the etiological agents of diarrhea among Danish children determined that rotavirus was the most frequent agent detected (15). The finding that *Granulicatella* was positively associated with diarrhea is novel for a well-nourished and breastfed cohort living in a high-income country, but it corroborates past reports of *Granulicatella* association with diarrhea among children in low-income countries and provides further evidence that diarrhea may provide the conditions for facultative anaerobes to expand in the gastrointestinal tract. Relatedly, the positive associations of Faith’s phylogenetic diversity and Staphylococcales abundance during the first 3.5 months of life with diarrhea later in infancy suggest that early infant gut microbiota should be investigated further to determine the contribution to future infant gastroenteritis.

### Limitations

The dates were not gathered for when infant morbidities occurred or for when medicine or supplements were administered to infants. Because of this, the health status and medicine/supplement use of infants at the time of stool sample collections are unknown, and the number of days elapsed between stool collection and morbidity or medicine/supplement use is unknown. Consequently, for some infants, the gut microbiome may have recovered from morbidity or medicine/supplement use before stool was collected, thereby diminishing or concealing the diagnostic signal in the study. It is also important to consider the possibility that some diarrhea cases may have been misdiagnosed by mothers because stool from exclusively breastfed infants tends to be mushy (70) and may have been difficult to distinguish from watery stool. Still, two well-documented associations—between stool alpha diversity and antibiotic use and between stool alpha diversity and diarrhea—were detected in this study and provide some support for the study design and power to detect associations between infant morbidity and the gut microbiome.

## Supplementary Material

Reviewer comments

## Data Availability

Bacterial 16S rRNA sequences are available from the NCBI Sequence Read Archive (www.ncbi.nlm.nih.gov/sra) under BioProject ID PRJNA1416402, experiment SRX31987205, run SRR37040161. Metadata associated with these sequences are available in Table S2. Requests for additional data from the Mother Infant Lactation Quality study used in this analysis should be made via an email to the senior USDA-ARS WHNRC author. Requests will be reviewed quarterly by a committee consisting of the study investigators.

## SUPPLEMENTAL MATERIAL

Supplemental Figures msystems.00134-26-s0001.pdf Fig. S1 to S10.

Table S1 msystems.00134-26-s0002.xlsx Statistical models used to determine relationships between the infant stool microbiota and morbidity.

Table S2 msystems.00134-26-s0003.xlsx Metadata descriptors of 16S rRNA amplicon sequence data.

OPEN PEER REVIEW reviewer-comments.pdf An accounting of the reviewer comments and feedback.

ASM does not own the copyrights to Supplemental Material that may be linked to, or accessed through, an article. The authors have granted ASM a non-exclusive, world-wide license to publish the Supplemental Material files. Please contact the corresponding author directly for reuse.
