# Supplementary material for: Stool microbial composition is associated with recent and future diarrhea and fever events in breastfed Danish infants
Source: mSystems. 2026 May 11;11(6):e00134-26. doi: 10.1128/msystems.00134-26 (PMC13288990; doi:10.1128/msystems.00134-26)
Supplement: Supplemental Figures — Fig. S1 to S10. [file msystems.00134-26-s0001.pdf]

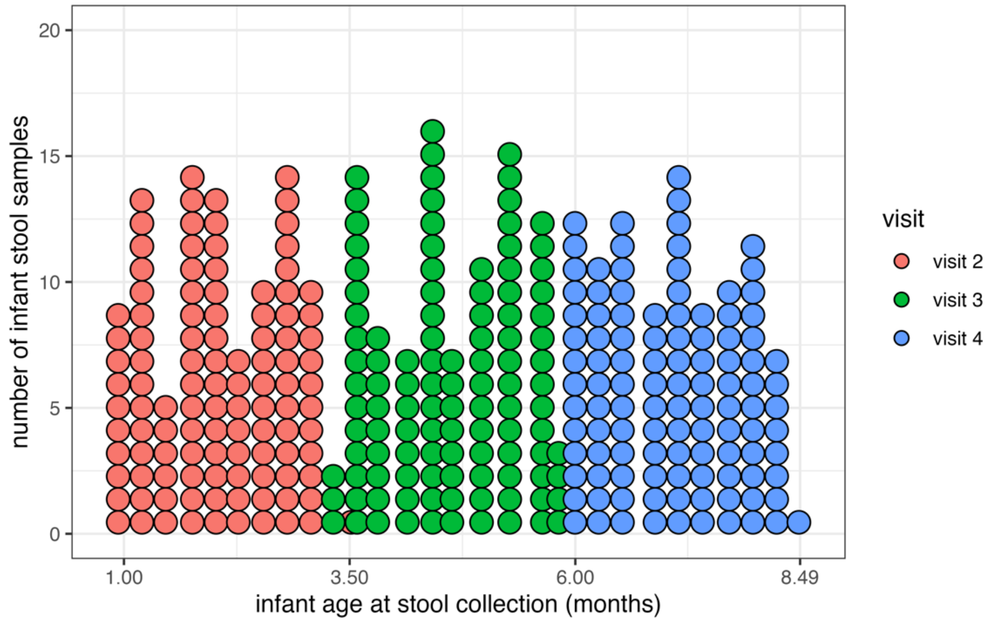

Figure S1. Distribution of infant age at time of stool collection in visits 2, 3, and 4.

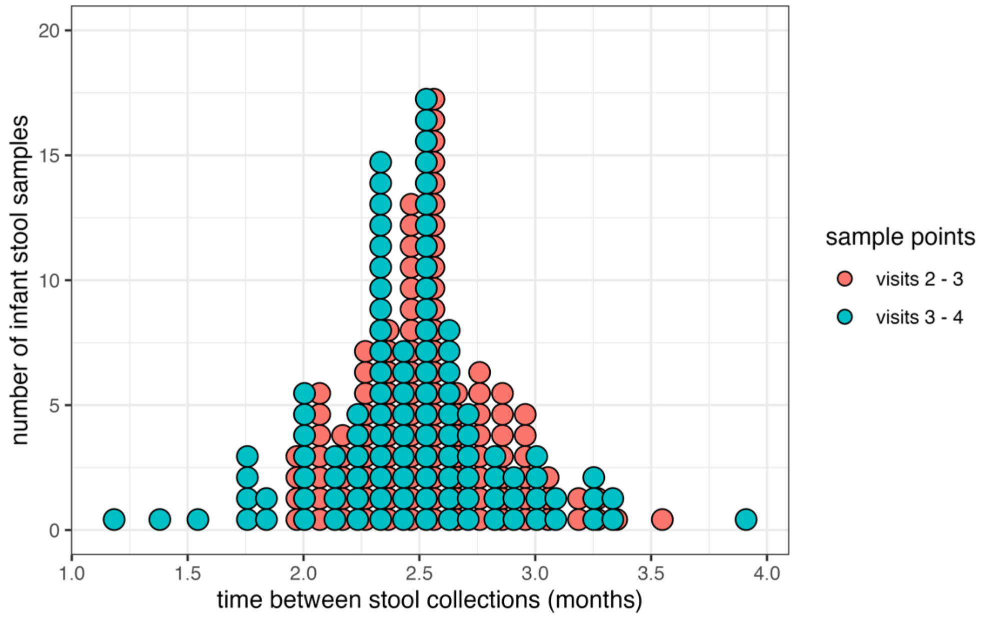

Figure S2. Distribution time (months) elapsed between visit 2 and visit 3 stool samples, and between visit 3 and visit 4 stool samples.

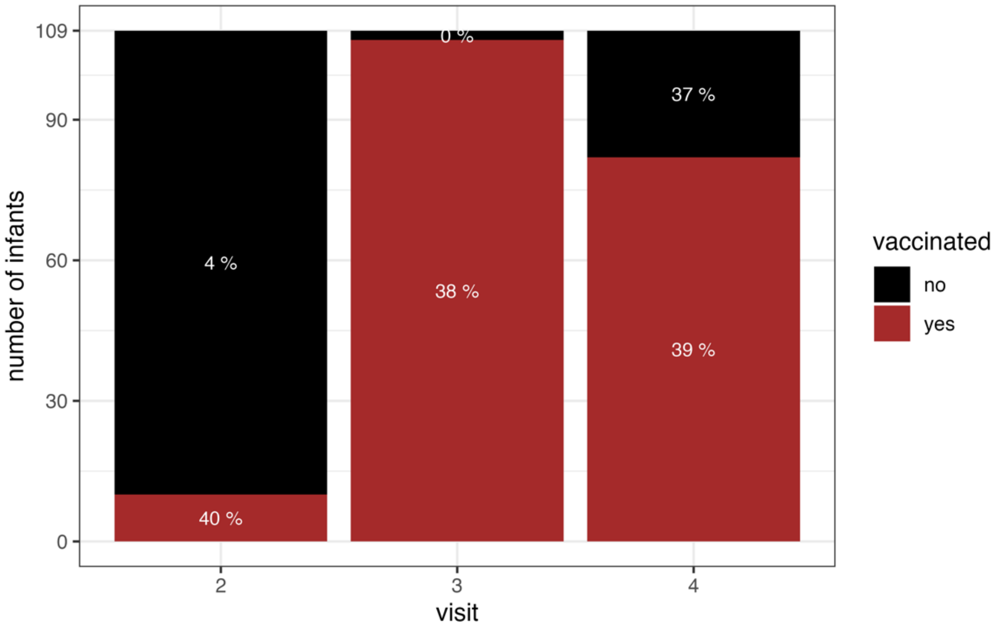

Figure S3. Stacked bar plot displaying total number of infants by vaccination status within each visit. The percentage of infants that developed fever in that visit period is printed on the bar illustrating their vaccination status.

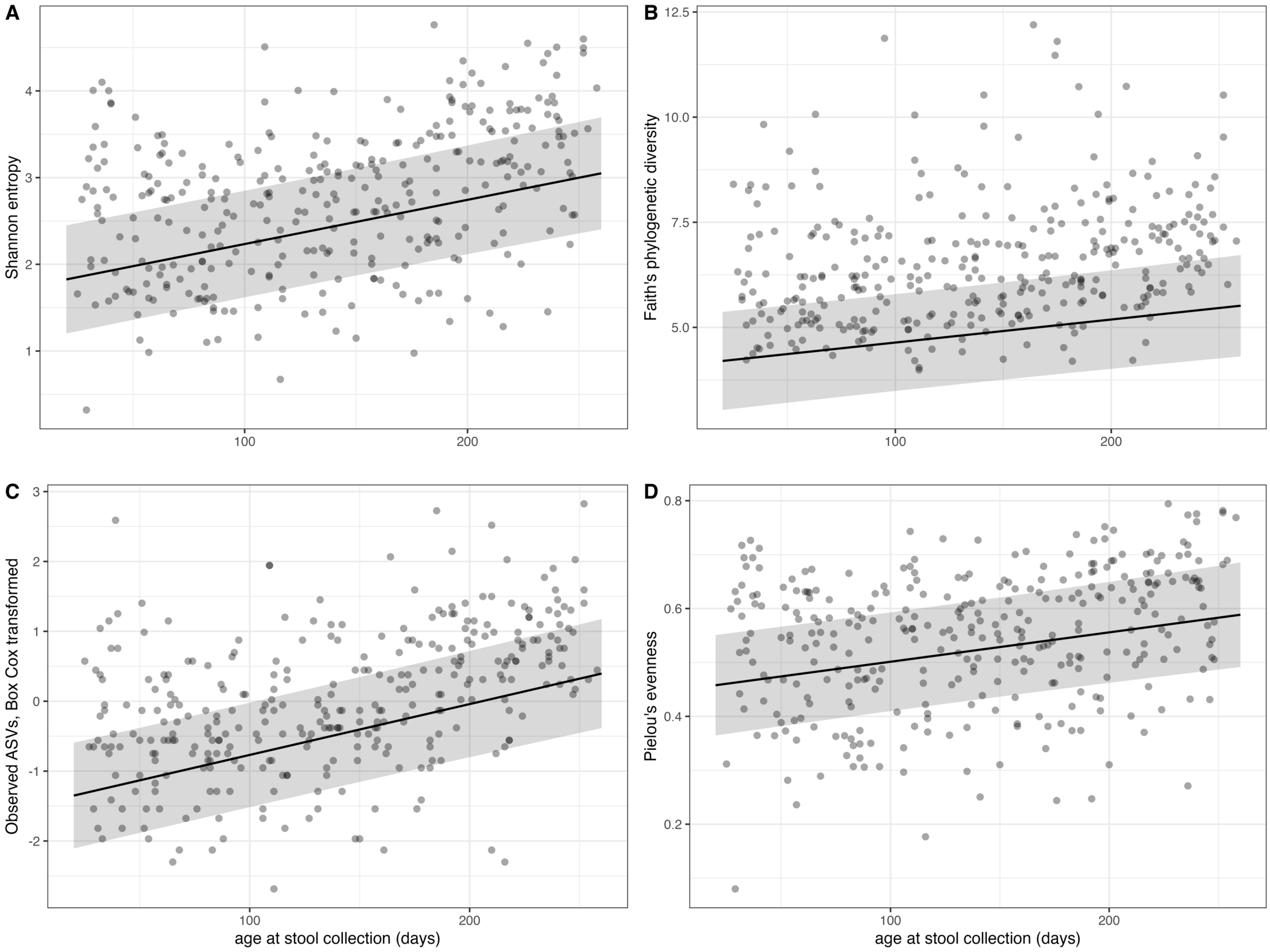

Figure S4. Infant stool microbial alpha diversity measures at all three sample timepoints for each individual. Shannon entropy (A), Faith's phylogenetic diversity (B), Box Cox-transformed observed ASVs (C), and Pielou's evenness (D) were positively associated with infant age in days. Raw data points are displayed, as well as the slope and 95 % confidence interval for the predicted values.

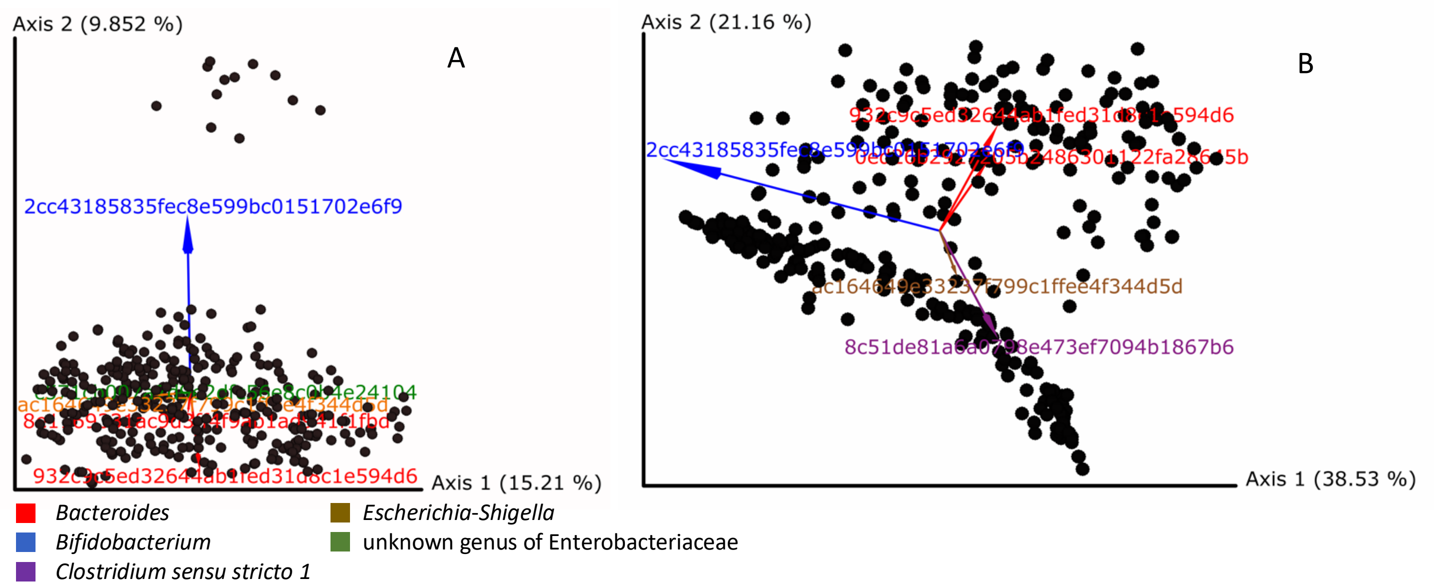

Figure S5. Principal coordinates analysis of unweighted (A) and weighted (B) UniFrac distances among infant stool samples. The five ASVs contributing most to variance in UniFrac distances are projected on the principal coordinates. Genus level taxonomic classification of ASVs are displayed in the figure key.

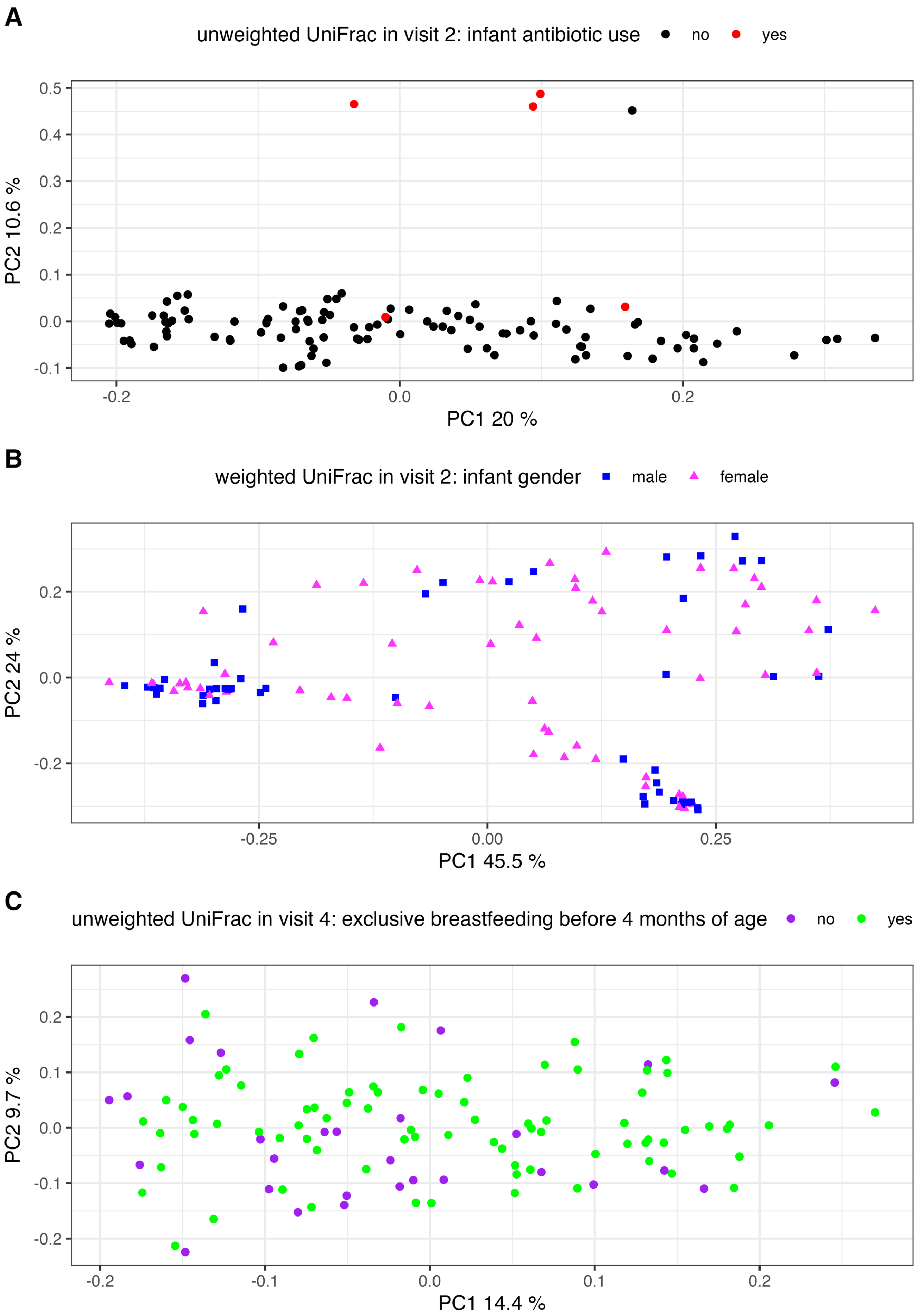
A
unweighted UniFrac in visit 2: infant antibiotic use
no
yes
PC2 10.6 %
PC1 20 %
B
weighted UniFrac in visit 2: infant gender
male
female
PC2 24 %
PC1 45.5 %
C
unweighted UniFrac in visit 4: exclusive breastfeeding before 4 months of age
no
yes
PC2 9.7 %
PC1 14.4 %

Figure S6. Principal coordinates analysis of unweighted and weighted UniFrac distances from cross-sectional data (i.e. single visit collection). Samples are distinguished by covariates that significantly associated with the UniFrac distances from that visit. (A) Unweighted UniFrac distances in stool samples from infants that received or did not receive antibiotics in the same visit. (B) Weighted UniFrac distances in visit 2 stool samples from infant males versus infant females. (C) Unweighted UniFrac distances in visit 4 stool samples from infants that were or were not exclusively breastfed the first 4 months of life.

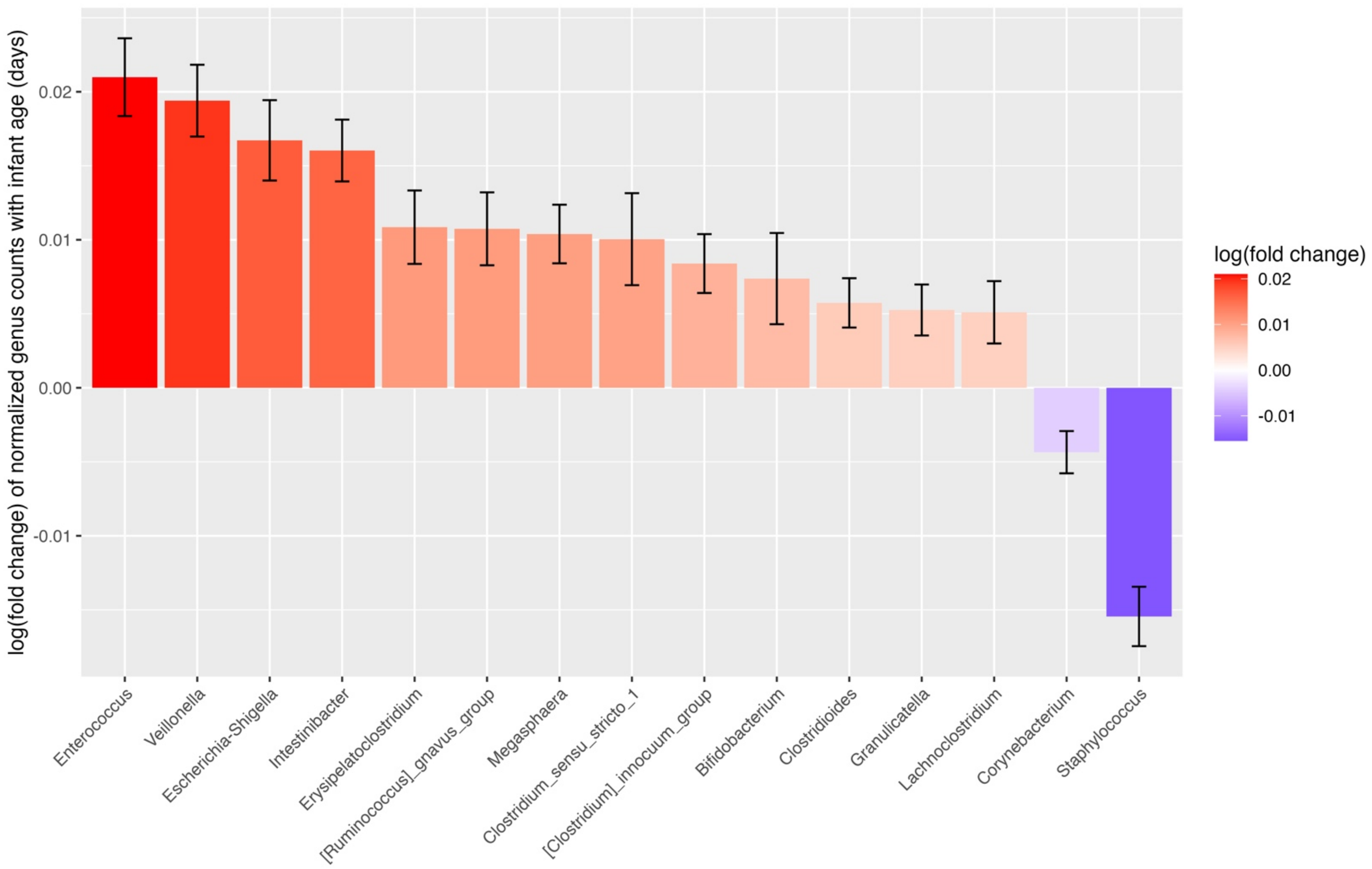

Figure S7. Bar plot of log(fold change) abundance of stool microbial genera with infant age (days), as determined using ANCOM-BC2. The standard error is also shown.

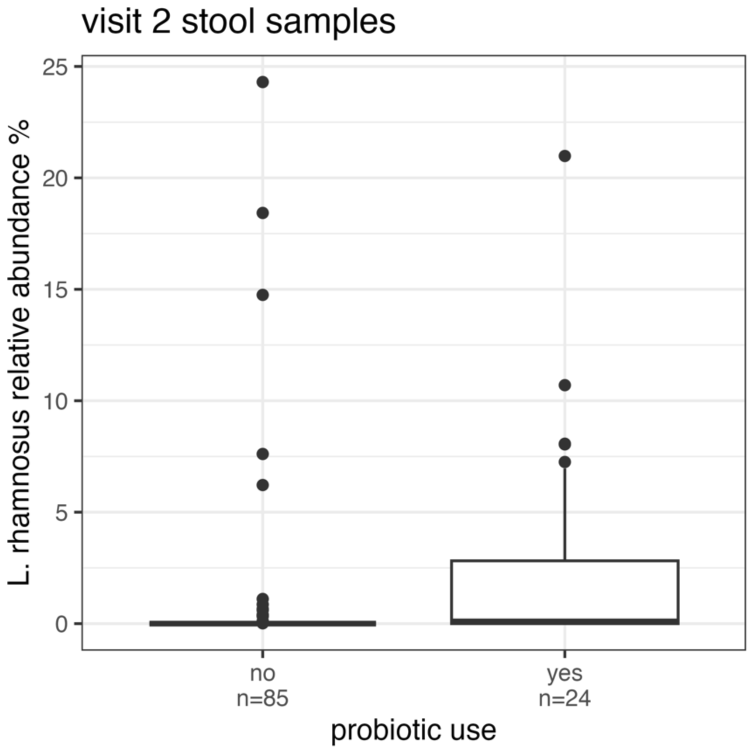

Figure S8. Box plot of *Lactobacillus rhamnosus* relative abundance (%, based on rarefied counts) in infant stool by infant probiotic use in visit 2.

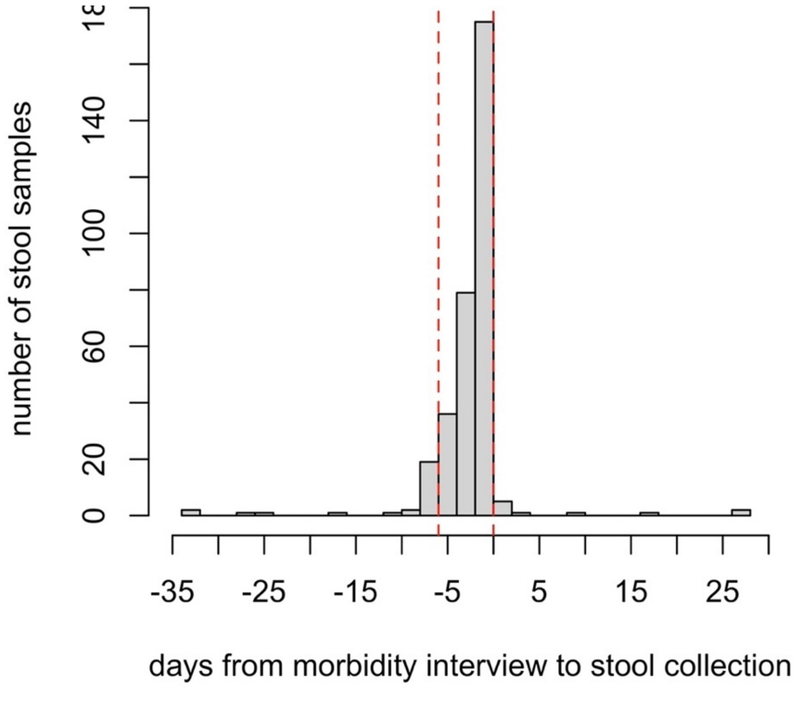

Figure S9. Histogram of days from morbidity interview to stool collection. Stool within red dashed lines were collected within the week prior to the morbidity interview.

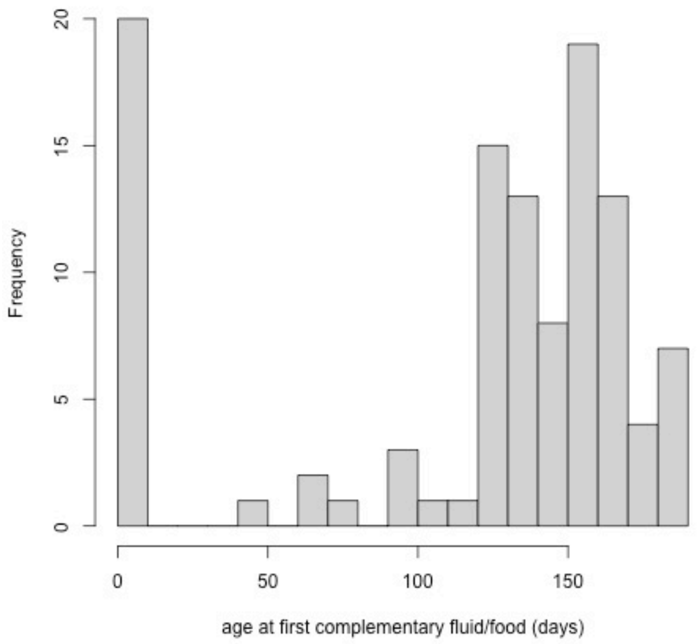

Figure S10. Histogram of infant age (days) at the time of introduction of complementary fluids or foods.

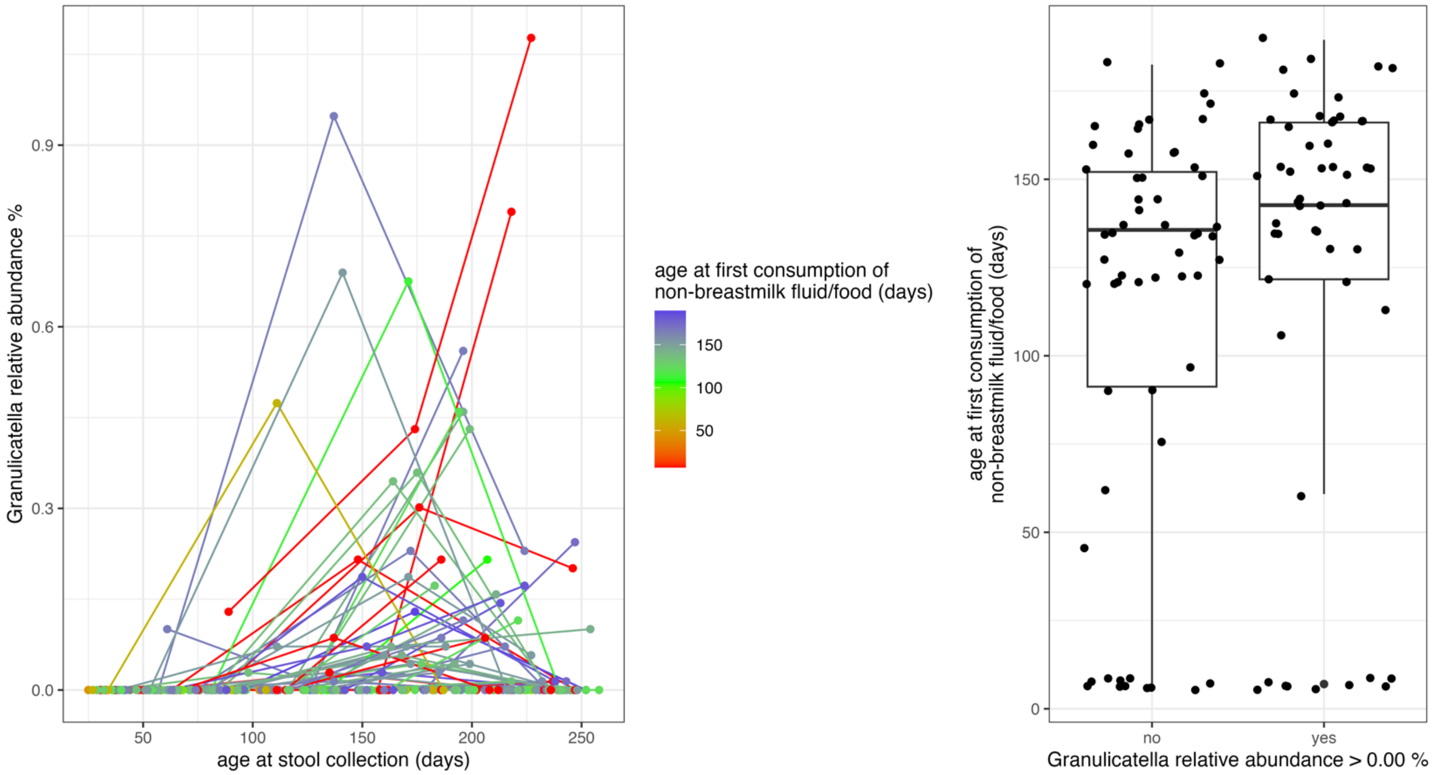

Figure S11. Scatterplot (A) of *Granulicatella* relative abundance at each stool collection with lines connecting stool samples from the same infant, and points and lines colored by infant age at introduction of fluid or food, in days. Box plot (B) of infant age at introduction of fluid or food, in days, for infants with stool samples containing 0.00 % *Granulicatella* relative abundance versus infants with at least one stool sample containing > 0.00 % *Granulicatella* relative abundance.
