# Supplementary material for: Stool microbial composition is associated with recent and future diarrhea and fever events in breastfed Danish infants
Source: mSystems. 2026 May 11;11(6):e00134-26. doi: 10.1128/msystems.00134-26 (PMC13288990; doi:10.1128/msystems.00134-26)
Supplement: Reviewer comments [file reviewer-comments.pdf]

AMERICAN SOCIETY FOR MICROBIOLOGY | mSystems®

Stool microbial composition is associated with recent and future diarrhea and fever events in breastfed Danish infants.

Laurynne Coates, David Storms, Sarah Spearman, Setareh Shahab-Ferdows, Sophie Chistensen, Jack Lewis, Christian Mølgaard, Kim Michaelsen, Lindsay Allen, Danielle Lemay, and Mary Kable

Corresponding Author(s): Mary Kable, USDA-ARS, Western Human Nutrition Research Center

Review Timeline:

Submission Date:	February 4, 2026
Editorial Decision:	March 9, 2026
Revision Received:	March 31, 2026
Accepted:	April 6, 2026

Editor: Fatima Pereira

Reviewer(s): Disclosure of reviewer identity is with reference to reviewer comments included in decision letter(s). The following individuals involved in review of your submission have agreed to reveal their identity: Calum J Walsh (Reviewer #1); Susheel Banu Busi (Reviewer #2)

Transaction Report:

(Note: With the exception of the correction of typographical or spelling errors that could be a source of ambiguity, letters and reports are not edited. The original formatting of letters and referee reports may not be reflected in this compilation.)

DOI: https://doi.org/10.1128/msystems.00134-26

Re: mSystems00134-26 (Stool microbial composition is associated with recent and future diarrhea and fever events in breastfed Danish infants.)

Dear Dr. Mary E. Kable:

Thank you for the privilege of reviewing your work. Below you will find my comments, instructions from the mSystems editorial office, and the reviewer comments.

-A search for PRJNA1416402 in the NCBI Sequence Read Archive returned no results. To comply with the ASM Open Data Policy (https://journals.asm.org/open-data-policy), please make the sequencing data publicly available immediately. Additionally, please ensure adherence to community metadata standards by providing a table containing minimum information about the deposited data (e.g. MIMARKS as described in DOI: 10.1038/nbt.1823.)

Marked-up manuscript:
- Line 111- alcohol consumption is per week, day? Please indicate.
- The discussion must be condensed and redundancies removed. For example, lines 565-568 state twice that Granulicatella has been identified in breast milk. Reduce or eliminate descriptions of Granulicatella spp that are neither relevant nor present in the gut.
- Please increase labels on Figure 1 (L, P, A) - these are not readable.
- Font size for axes labels/titles of figure 7 also need to be increased.

Please return the manuscript within 60 days; if you cannot complete the modification within this time period, please contact me. If you do not wish to modify the manuscript and prefer to submit it to another journal, notify me immediately so that the manuscript may be formally withdrawn from consideration by mSystems.

Revision Guidelines
To submit your modified manuscript, log into the submission site at https://msystems.msubmit.net/cgi-bin/main.plex. Go to Author Tasks and click the appropriate manuscript title to begin. The information you entered when you first submitted the paper will be displayed; update this as necessary. Note the following requirements:

• Upload point-by-point responses to the issues raised by the reviewers in a file named "Response to Reviewers," NOT in your cover letter.
• Upload a compare copy of the manuscript (without figures) as a "Marked-Up Manuscript" file.
• Upload a clean .DOC/.DOCX version of the revised manuscript and remove the previous version.
• Each figure must be uploaded as a separate, editable, high-resolution file (TIFF or EPS preferred), and any multipanel figures must be assembled into one file.
• Any supplemental material intended for posting by ASM should be uploaded with their legends separate from the main manuscript. You can combine all supplemental material into one file (preferred) or split it into a maximum of 10 files with all associated legends included.

For complete guidelines on revision requirements, see our Submission and Review Process webpage. Submission of a paper that does not conform to guidelines may delay acceptance of your manuscript.

Data availability: ASM policy requires that data be available to the public upon online posting of the article, so please verify all links to sequence records, if present, and make sure that each number retrieves the full record of the data. If a new accession number is not linked or a link is broken, provide mSystems production staff with the correct URL for the record. If the accession numbers for new data are not publicly accessible before the expected online posting of the article, publication may be delayed; please contact production staff (mSystems@asmusa.org) immediately with the expected release date.

Publication Fees: For information on publication fees and which article types are subject to charges, visit our website. If your manuscript is accepted for publication and any fees apply, you will be contacted separately about payment during the production process; please follow the instructions in that e-mail. Arrangements for payment must be made before your article is published.

ASM Membership: Corresponding authors may join or renew ASM membership to obtain discounts on publication fees. Need to upgrade your membership level? Please contact Customer Service at Service@asmusa.org.

The ASM Journals program strives for constant improvement in our submission and publication process. Please tell us how we can improve your experience by taking this quick Author Survey.

Thank you for submitting your paper to mSystems.

Sincerely,
Fatima Pereira
Editor
mSystems

Reviewer #1 (Comments for the Author):

The authors have thoroughly addressed all of my previous concerns. The additional data and clarifications have significantly strengthened the manuscript, and I believe the work is now much more robust. I am pleased to recommend this paper for publication.

Reviewer #2 (Comments for the Author):

Thank you for making the requested revisions.

Response to Reviewer Comments

Comments from the Editor:

-A search for PRJNA1416402 in the NCBI Sequence Read Archive returned no results. To comply with the ASM Open Data Policy (https://journals.asm.org/open-data-policy), please make the sequencing data publicly available immediately.

The repository has now been made public.

Additionally, please ensure adherence to community metadata standards by providing a table containing minimum information about the deposited data (e.g. MIMARKS as described in DOI: 10.1038/nbt.1823.)

Table S2 in supplementary file 3, now contains a mapping file like table including minimal information about the sequencing files and is referred to in the data availability portion of the manuscript.

Marked-up manuscript:

- Line 111- alcohol consumption is per week, day? Please indicate.

Thank you for catching this. The inclusion criterion for alcohol consumption was less than 50 mL per day. This has now been added to the manuscript where indicated.

- The discussion must be condensed and redundancies removed. For example, lines 565-568 state twice that Granulicatella has been identified in breast milk. Reduce or eliminate descriptions of Granulicatella spp that are neither relevant nor present in the gut.

We have now removed some of the material from the discussion section as well as sentences that were somewhat repetitive. We have also rearranged the paragraphs so that the type of illnesses (diarrhea vs fever) are next to each other, rather than the type of analyses (differential abundance vs. random forest modeling).

- Please increase labels on Figure 1 (L, P, A) - these are not readable.

We have now doubled the font size and set the font type as bold for the L, P, and A labels, to increase visibility.

- Font size for axes labels/titles of figure 7 also need to be increased.

We have increased the font size of all axes labels and axes/legend titles by 50% in Figure 7. We also shortened some of the label text to accommodate the increased size.

Reviewer #1

The authors have thoroughly addressed all of my previous concerns. The additional data and clarifications have significantly strengthened the manuscript, and I believe the work is now much more robust. I am pleased to recommend this paper for publication.

We appreciate the reviewer's thoughtful comments that helped greatly improve the manuscript.

Reviewer #2

Thank you for making the requested revisions.

Thank you for taking the time to review our manuscript and make thoughtful comments.

Re: mSystems00134-26R1 (Stool microbial composition is associated with recent and future diarrhea and fever events in breastfed Danish infants.)

Dear Dr. Mary E. Kable:

Your manuscript has been accepted, and I am forwarding it to the ASM production staff for publication. Your paper will first be checked to make sure all elements meet the technical requirements. ASM staff will contact you if anything needs to be revised before copyediting and production can begin. Otherwise, you will be notified when your proofs are ready to be viewed.

Data Availability: ASM policy requires that data be available to the public upon online posting of the article, so please verify all links to sequence records, if present, and make sure that each number retrieves the full record of the data. If a new accession number is not linked or a link is broken, provide production staff with the correct URL for the record. If the accession numbers for new data are not publicly accessible before the expected online posting of the article, publication may be delayed; please contact ASM production staff immediately with the expected release date.

Publication Fees: For information on publication fees and which article types have charges, please visit our website. We have partnered with Copyright Clearance Center (CCC) to collect author charges. If fees apply to your paper, you will receive a message from no-reply@copyright.com with further instructions. For questions related to paying charges through RightsLink, please contact CCC at ASM_Support@copyright.com or toll free at +1-877-622-5543. CCC makes every attempt to respond to all emails within 24 hours.

ASM Membership: Corresponding authors may join or renew ASM membership to obtain discounts on publication fees. Need to upgrade your membership level? Please contact Customer Service at Service@asmusa.org.

Embargo Policy: A press release may be issued as soon as the manuscript is posted on the mSystems Latest Articles webpage. The corresponding author will receive an email with the subject line "ASM Journals Author Services Notification" when the article is available online.

Cover Image Submissions: If you would like to submit a potential Cover Image, please email a file and a short legend to msystems@asmusa.org. Please note that we can only consider images that (i) the authors created or own and (ii) have not been previously published. By submitting, you agree that the image can be used under the same terms as the published article. Image File requirements: TIF/EPS, 7.5 inches wide by 8.25 inches tall (at least 2,250 pixels wide by 2,475 pixels tall), minimum 300 dpi resolution (600 dpi preferred), RGB, and no figure elements, e.g., arrows or panel labels. The legend should be a short description of the image, 1-2 sentences recommended. Please download and use this interactive template in Adobe to ensure that your proposed cover image meets our size requirements (https://journals.asm.org/pb-assets/pdf-text-excel-files/ASM-Interactive-Sizing-Cover-Template-1715689791.pdf).

Thank you for submitting your paper to mSystems.

Sincerely,
Fatima Pereira
Editor
mSystems
